# Identifying the 50 most productive researchers in top-tier, broad-scope educational psychology journals (2017–2022): a new perspective with a focus on publication trends and diversity

**DOI:** 10.3389/fpsyg.2025.1660783

**Published:** 2026-03-02

**Authors:** Veit Kubik, Lena Keller, Lea Jaspers, Annika Koch, Vincent Hoogerheide, Julian Roelle

**Affiliations:** 1 Department of Psychology IV, University of Würzburg, Würzburg, Germany; 2 Department of Educational Sciences, University of Potsdam, Potsdam, Germany; 3 Institute for Psychology of Learning and Instruction (IPL), Kiel University, Kiel, Germany; 4 Institute of Educational Science, Ruhr University Bochum, Bochum, Germany; 5 Department of Education, Utrecht University, Utrecht, Netherlands; 6 Institute for Psychology in Education and Instruction (IPBE), University Münster, Münster, Germany

**Keywords:** diversity, educational psychology, open science, productivity, publication trends, ranking

## Abstract

The present study identified the most productive researchers in educational psychology by analyzing the number of published articles in top-tier and broad-scope educational psychology journals from 2017 to 2022. Building on prior productivity research, we extended the scope of the analysis in three distinct ways: (1) we expanded the sample to include the 50 most productive researchers; (2) we applied three different scoring methods to assess productivity; and (3) we used a broader and more objectively defined set of journals, based on the Web of Science “Psychology, Educational” category. In addition, we conducted an online survey to examine characteristics of highly productive researchers and examined their publications in the target journals with respect to research topics, open science practices, collaboration patterns, and internationalization. Results indicated that three senior researchers (i.e., Richard E. Mayer, Reinhard Pekrun, and Herbert W. Marsh) consistently ranked among the top 5. In addition, early-career researchers accounted for a substantial share of the top 50, ranging from 25% to 40%. However, the diversity of the most productive researchers was limited: the majority identified as White (86%), male (59%), non-first-generation students (60%), and first-generation faculty members (86%), and primarily held European (46%) or North American nationalities (39%). Publication trends showed a predominance of quantitative studies, with articles typically reporting 1.2 studies and an average of 4.2 authors. The most frequently used keywords were motivation, quantitative methods, and multimedia learning, reflecting a broad range of research interests within the field.

## 1 Introduction

In many academic disciplines, research productivity is a key factor that influences whether researchers gain tenure, merit awards, and research funding (e.g., Győrffy et al., 2020). Accordingly, knowing which researchers are highly productive (as measured by the number of published articles in representative, top-tier journals; e.g., Smith et al., 1998) and which factors are associated with high productivity is of great interest to current and future generations of researchers. Therefore, there has been an increase in productivity studies in recent years (e.g., Flanigan et al., 2018; Fong et al., 2022; Prinz et al., 2020).

However, despite the recent focus on illuminating and understanding individual productivity in educational psychology, there are some points of criticism and blind spots in previous research on productivity that need to be addressed. First, in recent studies that quantified researchers' productivity by counting publications, there was no objective criterion for the selection of top-tier journals in the field of educational psychology (e.g., Fong et al., 2022; Greenbaum et al., 2016; Hsieh et al., 2004).

Second, past studies have focused on a relatively small number of researchers (i.e., the top 20 or 30) and, most importantly, provided little insight into the characteristics of these individuals. They did not assess key demographic and contextual factors such as age, gender, race/ethnicity, care responsibilities, or first-generation student and faculty status. Without such information, it is difficult to evaluate the degree of diversity within this group and, consequently, to understand whether high productivity emerges only under particular conditions or can be achieved by individuals facing a wider range of circumstances.

The present study, in which we examined individual productivity by analyzing articles published in top-tier and broad-scope educational psychology journals between 2017 and 2022, was designed to address these limitations and blind spots. We used an objective criterion to select top-tier journals (average ranking position ≤ 10 in the Web of Science “Psychology, Educational” category between 2017 and 2022), set a larger target number of highly productive researchers (i.e., the top 50), and conducted an online survey to determine the characteristics of this group of highly productive researchers. In line with prior ranking studies (e.g., Fong et al., 2022; Greenbaum et al., 2016; Hsieh et al., 2004), we conceptualized productivity as the number of articles published in these journals, which provides a consistent and comparable indicator of scholarly output, while acknowledging that this measure captures only one dimension of research productivity.

### 1.1 Prior research on productivity

Several studies have been published about research productivity in educational psychology between 1991 and 2021 (Fong et al., 2022; Greenbaum et al., 2016; Hsieh et al., 2004; Jones et al., 2010; Smith et al., 1998, 2003). In these studies, a “standard” set of journals have been repeatedly used to investigate individual and institutional productivity (i.e., *Contemporary Educational Psychology, Educational Psychologist, Educational Psychology Review*, and the *Journal of Educational Psychology*). In addition to this standard set of journals, the previous ranking studies included several other journals to examine productivity (e.g., *Cognition and Instruction*; see Table 1). These studies have shown that some highly productive researchers in educational psychology are publishing at a high rate in top-tier journals, in some cases even over several decades (e.g., Patricia A. Alexander, Herbert W. Marsh, and Richard E. Mayer).

**Table 1:** Characteristics of quantitative ranking studies.

**Period of assessment**	**Authors**	**Journals**	**Early career scholarsidentified**	**Scoring method**			**Individual productivity**	**Year of PhD**	**Background variables**				**Publication trends**			
				**Points**	**Count**	**Average**			**Proportion ofwomen**	**Race/ethnicity**	**Care-responsibility**	**First-generationstudent status**	**Collaboration** ^i^	**Internationalization**	**Article type**	**Topics**
**Individual productivity in general**																
1991–1996	Smith et al. (1998)	Standard^b^ + CI^c^	no	20	–	–	yes	no	no	no	no	no	no	no	yes	yes
1997–2001	Smith et al. (2003)	Standard^b^ + CI^c^	no	20	–	–	yes	no	no	no	no	no	no	yes	yes	yes
1991–2002	Hsieh et al. (2004)	Standard^b^ + CI^c^	no^g^	–	24	–	yes	yes	no	no	no	no	yes	no	no	no
2003–2008	Jones et al. (2010)	Standard^b^ + CI^c^	no^g^	25	32	–	yes	yes	no	no	no	no	yes	yes	no	no
2009–2014	Greenbaum et al. (2016)	Standard^b^ + CI^c^	no^g^	20	20	–	yes	yes	no	no	no	no	yes	yes	no	no
2015–2021	Fong et al. (2022)	Standard^b^ + CI^c, d^	yes	30	28	–	yes	yes	no	no	no	no	yes	yes	no	no
2017–2022	Present study	Standard^b^ + LI^e^ + LS^e^	yes	50	57	50	yes	yes	yes	yes	yes	yes	yes	yes	yes	yes
**Individual productivity with a focus on women**																
1976–1996^a^	Robinson et al. (1998)	Standard^b^ + JEE,^f^ AERJ^f^	no	–	yes^h^	–	no	no	yes	no	no	no	yes	no	no	no
1976–2004^a^	Evans et al. (2005)	Standard^b^ + JEE,^f^ AERJ^f^	no	–	yes^h^	–	no	no	yes	no	no	no	yes	no	no	no
2003–2008	Fong et al. (2009)	Standard^b^ + CI^c^	no	yes^h^	yes^h^	–	no	no	yes	no	no	no	yes	yes	no	no
2009–2016	Greenbaum et al. (2018)	Standard^b^ + CI^c^	no	23	23	–	yes	no	yes	no	no	no	yes	no	no	no
2017–2021	Griffin et al. (2023)	Standard^b^ + CI^c^	no	22	24	–	yes	yes	yes	no	no	no	yes	yes	no	no

–, not assessed.

^a^Data collected during even-number years.

^b^Journals additionally selected in the majority of productivity studies: Cognition and Instruction (CI).

^c^Fong et al. (2022) provided a supplemental analysis including articles published in Learning and Instruction, however, only for the count score.

^d^Journals additionally selected in the present study: Learning and Instruction (LI), and The Journal of the Learning Sciences (LS).

^e^Standard set of journals selected in all prior productivity studies: Educational Psychology Review (EPR), Educational Psychologist (EP), Journal of Educational Psychology (JEP), and Contemporary Educational Psychology (CEP).

^f^Journals additionally selected in Robinson et al. (1998) and Evans et al. (2005): Journal of Experimental Experimentation (JEE) and American Educational Research Journal (AERJ).

^g^Only the number of involved graduate students per article were provided.

^h^No absolute counts were reported because the analysis did not involve individual-level data but was based solely on the relative proportion of women in the field.

^i^Collaboration is measured as the average number of authors per article.

In addition to ranking studies that have examined researchers' productivity in educational psychology in general, there are also studies that have focused specifically on women's productivity in the field (Evans et al., 2005; Fong et al., 2009; Greenbaum et al., 2018; Griffin et al., 2023; Robinson et al., 1998). Furthermore, qualitative interviews have been conducted in follow-up studies with a small number of highly successful educational psychologists (Flanigan et al., 2018; Kiewra and Creswell, 2000; Kiewra et al., 2021; Patterson-Hazley and Kiewra, 2013) and specifically with selected highly productive female educational psychologists (Mayrath, 2008; Prinz et al., 2020). In these studies, researchers answered questions, for example, about important mentors and their strategies for successfully managing their time and research resources.

As a traditional continuation of previous rankings, the most recent study by Fong et al. (2022) identified the highly productive (early career) researchers and the top-producing institutions for the period from 2015 to 2021.

### 1.2 Limitations of prior studies on research productivity

One major limitation of prior research on productivity in educational psychology is the unsystematic selection of journals used to determine individual productivity. An analysis of the journals included in previous productivity studies shows that most studies (i.e., Fong et al., 2022; Greenbaum et al., 2016; Hsieh et al., 2004; Jones et al., 2010; Smith et al., 1998, 2003) examined articles published primarily in the four “standard” journals of educational psychology (i.e., *Educational Psychologist*, the *Journal of Educational Psychology, Educational Psychologist*, and *Educational Psychology Review*) and additionally in *Cognition and Instruction*. So far, the same set of journals has been selected to assess the productivity of researchers over time, without a convincing rationale as to why these five journals in particular were selected and other top-tier journals were not. One of the earlier productivity studies (Smith et al., 1998) described its rationale as follows:

Five journals considered to be among the “core journals” in the field and, thus, those in which educational psychologists are likely to publish their scholarship were examined… the journals we targeted provide a representative sampling of educational psychologists' work, as all are peer-reviewed, publish articles on a wide variety of topics of interest to educational psychologists, and contain many different kinds of papers (e.g., original research and meta-analyses, essays, literature reviews). These are among the journals most likely to be found on the bookshelves of educational psychologists. (pp. 173–175)

Fong et al. (2022) reported that these five journals were chosen as they have been selected in previous productivity studies (for a similar reasoning, see Greenbaum et al., 2018; Hsieh et al., 2004), and that they were “representative” (p. 2) journals within educational psychology (see also Griffin et al., 2023).

But what exactly do the authors mean by representative? In prior studies on productivity, no objective criteria were specified to identify representative journals in educational psychology. Smith et al. (1998) referred to Walberg and Haertel (1992), who regarded journals to be core journals when “identified in the 1988 Social Sciences Citation Index” as “those that are frequently cited by each other and by related journals from allied fields” (p. 13). Based on this criterion, Smith et al. (1998) selected the *Journal of Educational Psychology, Educational Psychologist*, and *Contemporary Educational Psychology*. Furthermore, they selected *Educational Psychology Review* and *Cognition and Instruction* (CI) because “CI has, arguably, become synonymous with work in the field of educational psychology” (p. 175) and “it seems reasonable to assume that many educational psychologists target their work for publication in these journals and read the contents of most of these journals” (p. 175).

This set of journals originally selected by Smith et al. (1998) became the “standard” set of journals for productivity studies, and has never been actively challenged or updated toward the ever-changing, increasingly international landscape of educational psychology journals. Thus, the journal selection criteria in previous ranking studies were unclear, and if available, outdated, failing to adequately reflect the selected journals' evolving status and quality. This begs the question of how these “Champions League Journals” (Alexander Renkl in Flanigan et al., 2018) should be characterized.

We argue that journals used to determine the productivity of researchers in educational psychology should be both high quality and broad in scope, and should be selected based on (relatively) objective and valid criteria for all journals in the field of educational psychology. In the present study, we defined top-tier journals as those journals that were among the top 10 journals in educational psychology based on their average rank in the Web of Science “Psychology, Educational” list from 2017 to 2022. This list ranks the impact and significance of journals within the field based on a variety of criteria, including editorial quality, international score, and citation counts. As the research landscape changes over time, not all of the previously included journals have maintained their status. For example, *Cognition and Instruction* has not been a part of the top 10 in the Web of Science “Psychology, Educational” list since 2019 and has an average rank of 17.17 between 2017 and 2022 (for a similar argument, see also Griffin et al., 2023). In contrast, *Learning and Instruction* and the *Journal of the Learning Sciences* have been top-tier journals for years, but have never been included in productivity rankings (e.g., Greenbaum et al., 2016; Hsieh et al., 2004; Jones et al., 2010; Smith et al., 1998, 2003). To date, the study by Fong et al. (2022) is the only study to include articles published in *Learning and Instruction*, which resulted in notable changes in the productivity ranking compared to the “standard” set of journals and *Cognition and Instruction*. However, no objective criterion was reported for the decision to include *Learning and Instruction*, and only one of two scoring methods was applied (i.e., the count-based method, see below). Thus, it is unclear why only this journal was added, and only one scoring method was used to rank the productivity with this new set of journals. In the present study, we defined broad-scope journals as those that cover a broad range of key topics within educational psychology and are not tied to specific subfields, populations, or applied contexts. For this reason, we excluded journals that target specific subfields within educational psychology (e.g., *Child Development*).

A second limitation of previous productivity studies is the way the number of published articles is scored, which also impacts the measurement of researchers' productivity. Early studies used different scoring methods to operationalize research productivity, resulting in partially divergent results (Hsieh et al., 2004). For example, Smith et al. (1998, 2003) selectively used a point-based score to measure research productivity, while Hsieh et al. (2004) used a count-based score. The count-based score is derived by simply counting the number of articles published, regardless of the author position (Hsieh et al., 2004). The point-based score takes author position into account, with higher positions resulting in more points awarded (Smith et al., 1998, 2003; for details, see Section 2). The two scores can be inconsistent, especially when researchers differ in the number of articles and/or their position in the author line. The point score penalizes researchers who work in large teams with many co-authors or with early career researchers who typically serve as the first author in collaborations. In contrast, the count score favors researchers with middle author positions and many collaborations, in which they might not have contributed as much. Recent ranking studies (e.g., Fong et al., 2022; Greenbaum et al., 2016; Jones et al., 2010) used both scoring methods and found differences in the ranking results for research productivity (e.g., the ranking order changed and new researchers entered the top 30 list of highly productive researchers, see Fong et al., 2022).

A third limitation is that prior research has provided little information about the background and diversity of highly productive researchers. This information would help the field better understand highly productive researchers and what strategies, work habits, and/or working conditions may have helped them to reach the top of the productivity rankings. At the same time, such insights could also stimulate discussions about which social groups (e.g., racial/ethnic, socioeconomic, gender) are (or are not) represented at the top of the productivity rankings and why this may be the case.

Fourth, prior research has typically identified a rather small sample of 20–30 productive researchers, which is unlikely to contribute to a diverse representation of individuals. A larger sample of highly productive researchers (a) is likely to provide a more holistic view of the field, (b) provides broader recognition of a greater number of individuals, and (c) is more likely to include and thus encourage emerging and early career researchers.

### 1.3 Aims of the present study

In this productivity study, we aimed to identify highly productive researchers based on their articles published in top-tier broad-scope journals in educational psychology between 2017 and 2022. To do so, we applied objective inclusion criteria (i.e., average ranking position within the top 10 in the Web of Science “Psychology, Educational” list from 2017 to 2022; see Section 2 for details). This 6-year window aligns with the time frames used in prior ranking studies and reflected the most recent complete publication period available when coding occurred in 2023. By targeting the top 50 most productive researchers, we aimed to achieve a more diverse sample of researchers. To acknowledge the controversy in measuring productivity, we determined the most productive researchers based on both scoring methods. Furthermore, we proposed and calculated a combined score based on the averaged point- and count-based scores to (partially) compensate for their individual shortcomings.

One important goal of this study is also to gain further insight into the characteristics of highly productive researchers in educational psychology. Indeed, the characteristics of the most productive researchers remain largely unexplored, because prior studies have, at most, assessed only singular background information (e.g., the year of PhD and their gender). To this end, we conducted an additional survey study to assess background characteristics (e.g., gender, ethnicity, and first-generation student status), working habits, and care responsibilities of the selected 50 most productive researchers. Women often carry a disproportionate share of domestic and caregiving responsibilities, including childcare, elder care, and other forms of family-related care work (e.g., Horne et al., 2018). Because balancing these responsibilities with the demands of a scientific career can be challenging and may affect women's productivity (e.g., Collins et al., 2021; Jolly et al., 2014), we paid particular attention to gender differences with respect to childbearing, division of care work, and work habits.

An additional goal was to explore general trends and developments in educational psychology, such as the degree to which researchers collaborate (as measured by the average number of co-authors) and the types of studies that are published (e.g., original studies, research syntheses). Furthermore, we analyzed the research topics of highly productive researchers, the number of studies per publication, and the adoption of open science practices.

## 2 Method

### 2.1 Identification of the top 50 most productive researchers

To analyze researchers' productivity in educational psychology, we examined individuals' number of published articles in all issues of the following top-tier, broad-scope educational psychology journals between 2017 and 2022: *Educational Psychology Review, Educational Psychologist, Journal of Educational Psychology, Learning and Instruction, Journal of the Learning Sciences*, and *Contemporary Educational Psychology*. By March 22, 2023, we arrived at this modified list of journals in educational psychology and identified the top 50 most productive researchers from 2017 to 2022 in the three steps detailed below.

As a first step, we determined top-tier journals in educational psychology by selecting the journals with an average rank of ≤ 10 on the Web of Science “Psychology, Educational” list between 2017 and 2022. This resulted in nine journals with an average ranking position of ≤ 10: *Educational Psychology Review* (average ranking position 1.33), *Educational Psychologist* (average ranking position 2.00), *Journal of Educational Psychology* (average ranking position 3.50), *Child Development* (average ranking position 4.33), *Learning and Instruction* (average ranking position 5.33), *Journal of Counseling Psychology* (average ranking position 7.50), *Journal of the Learning Sciences* (average ranking position 7.83), *Contemporary Educational Psychology* (ranking position 9.33), and *Journal of School Psychology* (average ranking position 9.83).

As a second step, we assessed the scope and aims of the journals to determine whether they could be considered broad in scope, that is, journals that cover all topics in educational psychology and that are not tied to specific thematic areas or populations. Based on this criterion of topic generality, three of these journals were excluded: *Child Development*, which focuses on research on child development; the *Journal of Counseling Psychology*, which publishes research in counseling psychology for client and non-client populations with a specific focus, for example, on optimizing their potential and enhancing their wellbeing; and the *Journal of School Psychology*, which centers on psychological and behavioral processes in school settings. The remaining six top-tier, broad-scope journals were *Educational Psychology Review, Educational Psychologist*, the *Journal of Educational Psychology, Learning and Instruction*, the *Journal of the Learning Sciences*, and *Contemporary Educational Psychology*.

As a third step, we coded all articles from 2017 to 2022 from the selected journals. In total, our database included 7170 articles and 4428 authors. Similar to prior research (e.g., Fong et al., 2022), we calculated productivity scores for each researcher using the count-based and point-based scoring method, resulting in partially overlapping but not identical lists. For the *count-based method*, we counted the number of articles published independently of the author's position (Hsieh et al., 2004). For example, Researcher A published one article as a first author (with one co-author), two articles as a second author (with two co-authors), and one article as a last author (with three co-authors). Based on the count system, Researcher A receives a score of 4 (i.e., 4 published articles) in total. Based on the point-system scoring, we calculate the credit of Researcher A with the formula of Howard et al. (1987): $credit=\frac{1.5^{n-i}}{\overset{n}{\underset{i=1}{\sum }}1.5^{i-1}}$, with *n* as the number of authors and *i* as the ordinal author position. For the first author publication (with one co-author), Researcher A receives a score of $\frac{1.5^{2-1}}{1.5^{1}+1.5^{0}}$ = 0.6 credits; as a second author (with two co-authors), Researcher A earns $\frac{1.5^{3-2}}{1.5^{2}+1.5^{1}+1.5^{0}}$ = 0.316 credits for each of the two published articles; for the last author publication (with three co-authors), Researcher A earns $\frac{1.5^{4-4}}{1.5^{3}+1.5^{2}+1.5^{1}+1.5^{0}}$ = 0.123 credits. In total, Researcher A receives 1.355 credits (1 × 0.6 + 2 × 0.316 + 1 × 0.123) using the point-based scoring method. We then identified the 50 most productive researchers based on the point-based and count-based scoring method^1^. Additionally, we created a third ranking list by averaging the ranks of the researchers obtained by the count- and point-based scoring methods. For example, Researcher A has a rank of 1 with the count-based scoring method and a rank of 5 based on the point-based scoring method. Based on the combined method, we averaged both ranks, resulting in a score of 3 (i.e., (1 + 5)/2). We then identified the 50 most productive researchers based on the combined method (i.e., the 50 researchers with the highest combined ranks).

### 2.2 Identification of publication trends for the top 50 most productive researchers

For all 7,170 identified articles, we extracted and coded the following information: publication year, author names, author position, institutional affiliations, and article type. Similar to prior research (e.g., Fong et al., 2022; Greenbaum et al., 2016; Jones et al., 2010), we included editorials (i.e., introductions to special issues), reviews, essays, and interviews, but not errata and in memoria articles. We coded different article/study types using the following a priori categories:

(1) *Experimental articles*: Publications that analyzed data based on newly collected data or existing data (e.g., secondary analyses of data from data repositories, such as the Open Science Framework [OSF]) and used an experimental or quasi-experimental design (i.e., manipulation of an independent variable).
(2) *Correlational article*: Publications that analyzed data based on newly collected data or existing data (e.g., secondary analyses of data from data repositories, such as OSF, or of public use files from large-scale assessments, such as the Programme for International Student Assessment) and used a correlational design (i.e., no experimental groups were reported).
(3) *Case study/interview study*: Publications that analyzed data based on newly collected data or existing data (e.g., secondary analyses of data from data repositories, such as OSF) and used a case study (i.e., a detailed examination of a single subject) or interview study design (i.e., a researcher conducts interviews with individuals).
(4) *Mixed method study*: Publications that analyzed data based on newly collected data or existing data (e.g., secondary analyses of data from data repositories, such as OSF) and combined quantitative and qualitative research methods.
(5) *Theoretical article*: Publications that targeted the theoretical advancement of the field.
(6) *Review articles*: Publications that provided a qualitative summary of prior research.
(7) *Meta-analysis with or without a systematic review*: Publications that provided a quantitative summary of prior research.
(8) *Method article*: Publications that described and evaluated new research methodologies, techniques, or tools, offering detailed insights and/or guidelines for their application.
(9) *Introduction to a special issue*
(10) *Commentary*: Publications that commented on other articles or reflected on the field.
(11) *Book review*
(12) *Other*: Category for articles that we could not classify into the above-mentioned categories.

Illustrative example publications for each study type are provided in Supplementary Table S1.1 of the online supplement (OS).

Furthermore, we coded information about the number of first-, last-, and sole-authored articles, number of studies per publication (for original studies), and the degree to which open science practices were used by counting the number of preregistrations and registered reports for all researchers in the rankings.

Based on the frequency of the articles' keywords, we identified up to three descriptors per researcher that best represented the primary topic on which they had published (e.g., self-regulated learning, example-based learning, and multimedia learning). For each researcher, we then calculated the relative percentage of each descriptor using the formula:


$$\begin{array}{l} \frac{number\;of\;keywords\;of\;an\;individual\;descriptor\;}{number\;of\;articles\;published}\;\times \;100 \end{array}$$

For example, for Researcher A, who published four articles, we calculated the relative proportion of research topic from two keywords related to self-regulated learning (2/4 × 100= 50%), three keywords related to example-based learning (3/4 × 100= 75%), and one keyword related to multimedia learning (1/4 × 100= 25%). To derive these descriptors, semantically related keywords were grouped into broader topic clusters when such grouping was necessary. For instance, keywords such as *immersion, immersive virtual reality, virtual field trip*, and *virtual reality* were consolidated under the broader descriptor “*virtual reality*.” One author conducted the initial semantic grouping, and a second author reviewed the proposed clusters for conceptual consistency. Any discrepancies were discussed until agreement was reached.

Lastly, as an indicator of the degree of collaboration, we calculated the average number of authors for each article.

### 2.3 Exploring the background characteristics, working habits, and working conditions of the top 50 most productive researchers

To describe the sample with regards to background characteristics (i.e., diversity), working habits, and working conditions, we contacted individuals who appeared in any of the three lists and sent them an individual link to an online survey using the Qualtrics software (Qualtrics, 2023; a copy of the online survey is available in the OSF repository: https://doi.org/10.17605/OSF.IO/DWQFX). Because these lists were not identical and partially overlapped, they included *N* = 74 researchers, of whom 71 responded (however, the number of responses varied by question).

After providing informed consent, participants were asked to provide background information (e.g., age, gender, nationality, and ethnicity), academic background information (e.g., year of PhD, years in science, main PhD supervisor(s), academic position, first-generation student status, number of work hours per week, number of PhD students and PostDocs in total), and information about family and household commitment (e.g., number of hours for house work and/or care, nursing or custodial work; for all measured variables, see Supplementary Table S1.2).

The study was approved by the ethics committee of Ruhr University Bochum (EPE-2022–037) and informed consent was obtained from all participants. Participants had the opportunity to complete the survey from February 20 to June 26, 2023 and received up to three reminders, after which we stopped data collection. Because three researchers did not participate in the survey, we obtained basic information for them from Google Scholar, ORCID, ResearchGate, and/or university websites.

## 3 Results

### 3.1 Individual productivity

Tables 2–4 display the most productive researchers from 2017 to 2022 using the count-based, point-based, and combined method, respectively. The point-based method identified 50 highly productive researchers, the count-based method resulted in a list of 57 highly productive researchers (because 13 researchers shared rank 45), and the combined method resulted in a list of 50 highly productive researchers. The individual productivity results show that mostly senior researchers were ranked highest. In particular, three researchers appeared in the top 5 in all three rankings (i.e., Richard E. Mayer, Reinhard Pekrun, and Herbert W. Marsh) and one additional researcher appeared in the top 5 on only two of the three lists (i.e., Andrew J. Martin). These four researchers were also listed in prior ranking studies and thereby have proven to be highly productive researchers in the field of educational psychology over the last decades. Notably, a substantial number of early career researchers (here defined as those who received their doctorate in 2013 or later) have emerged as a new generation of top-performing early career researchers who were not included in prior rankings (except for the recent ranking by Fong et al., 2022). Among the most productive researchers, 40% were early career researchers in the point-based ranking, 25% in the count-based ranking, and 37% in the combined ranking. For example, Rebecca J. Collie, Logan Fiorella, Hanna Gaspard, and Vincent Hoogerheide appeared on all three lists of the present ranking study.

**Table 2:** Individual productivity of the top 50 researchers in top-tier and broad-scope educational psychology journals from 2017 to 2022 using the point-based method.

**Rank**	**Name**	**Points**	**Points per publication**	**Fong et al. (2022)**	**Greenbaum et al. (2016)**	**Jones et al. (2010)**	**Smith et al. (2003)**	**Smith et al. (1998)**
1	Mayer, Richard E.	10.665	0.355	1	14	1	1	2
2	Alexander, Patricia A.	7.632	0.636	2	17	2	9	8
3	Pekrun, Reinhard	7.058	0.243	11	17	–	–	–
4	Martin, Andrew J.	6.992	0.350	5	1	19	–	–
5	Marsh, Herbert W.	6.691	0.216	8	6	4	5	1
6	Wentzel, Kathryn R.	5.794	0.644	4	–	–	–	–
7	Fiorella, Logan^*^	5.680	0.437	3	–	–	–	–
8	Rau, Martina A.^*^	5.258	0.657	6	–	–	–	–
9	Bernacki, Matthew L.	5.168	0.345	15	–	–	–	–
10	Graham, Steve	4.971	0.552	7	–	6	3	–
11	Greene, Jeffrey A.	4.849	0.323	9	4	–	–	–
12	Collie, Rebecca J.^*^	4.835	0.302	29	–	–	–	–
13	Parker, Philip D.	4.758	0.216	–	–	–	–	–
14	List, Alexandra^*^	4.489	0.561	–	–	–	–	–
15	Roelle, Julian	4.476	0.407	30	–	–	–	–
16	Möller, Jens	4.460	0.165	–	–	–	–	–
17	Barzilai, Sarit	4.379	0.547	–	–	–	–	–
18	Lüdtke, Oliver	3.988	0.181	24	16	–	–	–
19	Kim, Young-Suk Grace	3.978	0.568	13	–	–	–	–
20	Sweller, John	3.921	0.261	–	10	17	–	–
21	Wolff, Fabian^*^	3.877	0.431	–	–	–	–	–
22	Ching, Boby Ho-Hong^*^	3.815	0.636	–	–	–	–	–
23	Lazarides, Rebecca^*^	3.809	0.476	–	–	–	–	–
24	Gaspard, Hanna^*^	3.795	0.316	–	–	–	–	–
25	Wigfield, Allan	3.708	0.309	19	–	–	11	–
26	Muis, Krista R.	3.667	0.306	12	–	–	–	–
27	Lindner, Marlit Annalena^*^	3.607	0.451	–	–	–	–	–
28	Becker, Michael	3.603	0.328	22	–	–	–	–
29	Rawson, Katherine A.	3.565	0.324	10	–	–	–	–
30	Guay, Frédéric	3.465	0.433	–	–	–	–	–
31	Schneider, Sascha^*^	3.430	0.312	–	–	–	–	–
32	Lauermann, Fani^*^	3.397	0.425	–	–	–	–	–
33	Flanigan, Abraham E.	3.375	0.422	–	–	–	–	–
34	Lachner, Andreas^*^	3.373	0.337	–	–	–	–	–
35	Schunn, Christian D.	3.369	0.374	18	–	–	–	–
36	Rutherford, Teomara^*^	3.347	0.669	–	–	–	–	–
37	Zhang, Xiao	3.278	0.410	–	–	–	–	–
38	Burns, Emma C.^*^	3.238	0.324	–	–	–	–	–
39	Dumas, Denis^*^	3.191	0.638	25	–	–	–	–
40	Scheiter, Katharina	3.157	0.243	–	–	–	–	–
41	Morin, Alexandre J. S.	3.110	0.239	–	–	–	–	–
42	Sinatra, Gale M.	3.109	0.346	–	–	16	–	–
43	Geary, David C.	3.067	0.340	–	–	–	–	–
44	Hoogerheide, Vincent^*^	3.031	0.276	–	–	–	–	–
45	Bardach, Lisa^*^	3.027	0.378	–	–	–	–	–
46	Guo, Jiesi^*^	3.009	0.158	–	–	–	–	–
47	Trautwein, Ulrich	3.007	0.100	–	13	13	–	–
48	Rosenzweig, Emily Q.^*^	2.920	0.417	–	–	–	–	–
49	Schüler, Anne	2.916	0.729	–	–	–	–	–
50	Graham, Sandra	2.874	0.718	–	–	–	–	–

The flags represent the countries where the researchers were employed at the time of the survey.

^*^Early career researchers who received their doctorate in 2013 or later.

**Table 3:** Individual productivity of the top 50 researchers in top-tier and broad-scope educational psychology journals from 2017 to 2022 measured by the count-based method.

**Rank**	**Name**	**Count**	**Fong et al. (2022)**	**Greenbaum et al. (2016)**	**Jones et al. (2010)**	**Hsieh et al. (2004)**
1	Marsh, Herbert W.	31	6	3	2	2
2	Mayer, Richard E.	30	1	9	1	1
2	Trautwein, Ulrich	30	2	4	3	–
4	Pekrun, Reinhard	29	9	9	–	–
5	Möller, Jens	27	8	–	–	–
6	Parker, Philip D.	22	11	–	–	–
6	Lüdtke, Oliver	22	4	2	3	–
8	Martin, Andrew J.	20	5	8	–	–
9	Guo, Jiesi^*^	19	–	–	–	–
10	van Gog, Tamara	18	7	4	–	–
11	Nagengast, Benjamin	17	12	12	–	–
11	Paas, Fred	17	3	1	20	–
13	Dicke, Theresa^*^	16	–	–	–	–
13	Collie, Rebecca J.^*^	16	27	–	–	–
15	Bernacki, Matthew L.	15	25	–	–	–
15	Eccles, Jacquelynne S.	15	–	–	–	–
15	Greene, Jeffrey A.	15	14	–	–	–
15	Sweller, John	15	16	12	10	12
19	Fiorella, Logan^*^	13	10	–	–	–
19	Goetz, Thomas	13	21	9	–	–
19	Mainhard, Tim	13	–	–	–	–
19	Morin, Alexandre J. S.	13	–	12	–	–
19	Scheiter, Katharina	13	–	–	–	–
24	Alexander, Patricia A.	12	13	–	6	7
24	Gaspard, Hanna^*^	12	–	–	–	–
24	Muis, Krista R.	12	17	–	–	–
24	Renkl, Alexander	12	–	4	14	–
24	Wigfield, Allan	12	18	–	–	12
24	Rey, Günter Daniel	12	–	–	–	–
30	Becker, Michael	11	19	–	–	–
30	Hoogerheide, Vincent^*^	11	–	–	–	–
30	Jansen, Malte^*^	11	15	–	–	–
30	Rawson, Katherine A.	11	24	–	–	–
30	Roelle, Julian	11	–	–	–	–
30	Göllner, Richard^*^	11	–	–	–	–
30	Schneider, Sascha^*^	11	–	–	–	–
37	Burns, Emma C.^*^	10	–	–	–	–
37	Klusmann, Uta	10	–	–	–	–
37	Lachner, Andreas^*^	10	–	–	–	–
37	Linnenbrink-Garcia, Lisa	10	20	–	–	–
37	Murayama, Kou	10	–	–	–	–
37	Preckel, Franzis	10	–	–	–	–
37	Robinson, Kristy A.^*^	10	–	–	–	–
37	Beege, Maik^*^	10	–	–	–	–
45	Fuchs, Lynn S.	9	–	4	6	–
45	Geary, David C.	9	–	–	–	–
45	Graham, Steve	9	23	–	3	3
45	Köller, Olaf	9	–	–	–	–
45	Nebel, Steve^*^	9	–	–	–	–
45	Roberts, Greg	9	–	–	–	–
45	Salmela-Aro, Katariina	9	–	–	–	–
45	Schunn, Christian D.	9	26	–	–	–
45	Sinatra, Gale M.	9	–	–	–	–
45	Bråten, Ivar	9	–	–	–	–
45	McBride, Catherine	9	–	–	–	–
45	Wolff, Fabian^*^	9	–	–	–	–
45	Wentzel, Kathryn R.	9	22	–	–	18

The flags represent the countries where the researchers were employed at the time of the survey.

^*^Early career researchers who received their doctorate in 2013 or later.

**Table 4:** Individual productivity of the top 50 most productive researchers in top-tier and broad-scope educational psychology journals from 2017 to 2022 measured by the combined point and count method.

**Rank**	**Name**	**Mean count & point rank**
1	Mayer, Richard E.	1.5
2	Marsh, Herbert W.	3
3	Pekrun, Reinhard	3.5
4	Martin, Andrew J.	6
5	Parker, Philip D.	9.5
6	Möller, Jens	10.5
7	Bernacki, Matthew L.	12
7	Lüdtke, Oliver	12
9	Collie, Rebecca J.^*^	12.5
10	Alexander, Patricia A.	13
10	Fiorella, Logan^*^	13
10	Greene, Jeffrey A.	13
13	Sweller, John	17.5
14	Roelle, Julian	22.5
15	Gaspard, Hanna^*^	24
16	Trautwein, Ulrich	24.5
16	Wigfield, Allan	24.5
18	Muis, Krista R.	25
19	Wentzel, Kathryn R.	25.5
20	Guo, Jiesi^*^	27.5
20	Graham, Steve	27.5
22	Becker, Michael	29
23	Rawson, Katherine A.	29.5
23	Scheiter, Katharina	29.5
25	Morin, Alexandre J. S.	30
26	Schneider, Sascha^*^	30.5
27	van Gog, Tamara	31
28	Rau, Martina A.^*^	33
28	Wolff, Fabian^*^	33
30	Lachner, Andreas^*^	35.5
31	Eccles, Jacquelynne S.	36
31	List, Alexandra^*^	36
33	Hoogerheide, Vincent^*^	37
34	Barzilai, Sarit	37.5
34	Burns, Emma C.^*^	37.5
36	Goetz, Thomas	39
37	Schunn, Christian D.	40
38	Lazarides, Rebecca^*^	40.5
39	Paas, Fred	41
40	Jansen, Malte^*^	41.5
40	Mainhard, Tim	41.5
42	Lindner, Marlit Annalena^*^	42.5
43	Guay, Frédéric	43.5
43	Sinatra, Gale M.	43.5
45	Geary, David C.	44
46	Lauermann, Fani^*^	45
47	Dicke, Theresa^*^	45.5
47	Flanigan, Abraham E.^*^	45.5
49	Robinson, Kristy A.^*^	47.5
49	Zhang, Xiao	47.5

The flags represent the countries where the researchers were employed at the time of the survey.

^*^Early career researchers who received their doctorate in 2013 or later.

In general, the three scoring methods resulted in overlap in the researchers listed, such that 33 researchers (45%) were listed in all three rankings and 17 researchers (23%) were listed in two of the three rankings. However, there were 24 researchers (32%) who appeared in only one of the rankings. Overall, the rankings based on the different scoring methods deviated from each other, suggesting that researchers may employ different publication strategies.

### 3.2 Publication trends

#### 3.2.1 Type of article/study

Most of the studies published by highly productive researchers during the review period were correlational studies, followed by experimental studies with an experimental study design (see Table 5 for information on the type of studies conducted by individual researchers). Review articles, meta-analyses, and theoretical articles made up a smaller proportion of published studies. Further details can be found in Table 6.

**Table 5:** Overview of publication trends: Author position, type of study, and number of preregistered studies among most productive researchers in top-tier and broad-scope educational psychology journals from 2017 to 2022.

**Name**	**Author position: Sole–first–last**	**Experimental article (average number of studies per publication)**	**Correlational article (average number of studies per publication)**	**Theoretical article**	**Review article**	**Meta-analysis and/or systematic review**	**Method article**	**Introduction to SI/commentary**	**Other^a^**	**Pre-registered studies**
Alexander, Patricia A.	5–0–5	1 (1.0)	2 (1.0)	1	1	0	2	2	0	0
Bardach, Lisa	0–6–0	0	4 (1.0)	0	2	1	1	0	0	0
Barzilai, Sarit	1–5–0	0	4 (1.2)	2	1	0	0	1	0	0
Becker, Michael	0–3–2	0	11 (1.0)	0	0	0	0	0	0	0
Beege, Maik^*^	0–2–0	7 (2.3)	0	0	1	2	0	0	0	0
Bernacki, Matthew L.	0–6–3	5 (1.2)	7 (1.0)	0	1	0	0	1	0	0
Bråten, Ivar	0–2–3	3 (1.0)	2 (1.0)	1	3	0	0	0	0	0
Burns, Emma C.^*^	0–5–0	0	10 (1.0)	0	0	0	0	0	0	0
Ching, Boby Ho-Hong^*^	1–5–0	1 (1.0)	5 (1.0)	0	0	0	0	0	0	0
Collie, Rebecca J.^*^	1–2–4	0	16 (1.1)	0	0	0	0	0	0	0
Dicke, Theresa^*^	0–3–5	0	15 (1.1)	0	0	0	1	0	0	0
Dumas, Denis^*^	2–2–0	0	3 (1.0)	0	1	0	1	0	0	0
Eccles, Jacquelynne S.	0–1–10	1 (1.0)	11 (1.0)	3	0	0	0	0	0	0
Fiorella, Logan^*^	1–6–2	10 (1.6)	0	0	3	0	0	0	0	0
Flanigan, Abraham E.^*^	1–3–1	0	2 (1.5)	1	0	0	0	0	5	0
Fuchs, Lynn S.	0–2–3	3 (1.0)	6 (1.0)	0	0	0	0	0	0	0
Gaspard, Hanna^*^	0–5–1	2 (1.0)	10 (1.0)	0	0	0	0	0	0	2
Geary, David C.	0–6–0	1 (1.0)	6 (1.0)	1	0	0	0	1	0	0
Goetz, Thomas	0–2–2	0	11 (1.1)	0	0	1	1	0	0	0
Göllner, Richard	0–1–3	2 (1.0)	7 (1.3)	0	0	0	2	0	0	0
Graham, Sandra	2–1–1	0	1 (1.0)	1	2	0	0	0	0	0
Graham, Steve	3–2–2	3 (1.0)	1 (1.0)	0	1	2	1	1	0	0
Greene, Jeffrey A.	1–4–4	2 (1.0)	4 (1.0)	1	2	2	0	1	1	0
Guay, Frédéric	1–4–1	2 (1.5)	5 (1.0)	0	0	0	0	0	0	0
Guo, Jiesi^*^	0–4–1	0	19 (1.1)	0	0	0	0	0	0	0
Hoogerheide, Vincent^*^	0–3–2	8 (1.6)	0	0	3	0	0	0	0	0
Jansen, Malte^*^	0–3–1	1 (5.0)	9 (1.0)	0	0	0	1	0	0	0
Kim, Young-Suk Grace	1–6–0	1 (1.0)	6 (1.0)	0	0	0	0	0	0	0
Klusmann, Uta	0–1–2	0	8 (1.2)	0	1	1	0	0	0	0
Köller, Olaf	0–0–8	4 (1.0)	4 (1.2)	0	0	0	1	0	0	0
Lachner, Andreas^*^	0–4–0	7 (1.4)	2 (1.0)	0	1	0	0	0	0	1
Lauermann, Fani^*^	0–4–2	0	5 (1.0)	0	1	1	0	1	0	0
Lazarides, Rebecca^*^	0–8–0	0	8 (1.0)	0	0	0	0	0	0	0
Lindner, Marlit Annalena^*^	1–4–2	7 (1.0)	0	0	0	1	0	0	0	1
Linnenbrink-Garcia, Lisa	0–2–8	0	8 (1.2)	0	1	0	1	0	0	0
List, Alexandra^*^	1–5–2	1 (1.0)	4 (1.5)	0	0	0	2	1	0	0
Lüdtke, Oliver	0–0–7	1 (1.0)	20 (1.2)	0	0	0	1	0	0	0
Mainhard, Tim	0–1–6	1 (1.0)	12 (1.0)	0	0	0	0	0	0	0
Marsh, Herbert W.	0–5–4	1 (1.0)	27 (1.0)	1	0	1	1	0	0	0
Martin, Andrew J.	0–8–4	0	20 (1.1)	0	0	0	0	0	0	0
Mayer, Richard E.	4–0–17	23 (1.8)	2 (1.0)	3	1	1	0	0	0	0
McBride, Catherine	0–0–6	1 (1.0)	6 (1.2)	0	1	1	0	0	0	0
Möller, Jens	0–0–21	4 (3.5)	19 (1.1)	1	0	1	2	0	0	0
Morin, Alexandre J. S.	0–0–4	1 (1.0)	12 (1.1)	0	0	0	0	0	0	0
Muis, Krista R.	0–3–1	5 (1.2)	5 (1.6)	2	0	0	0	0	0	0
Murayama, Kou	0–1–3	0	8 (1.1)	1	0	1	0	0	0	1
Nagengast, Benjamin	0–0–6	2 (1.0)	15 (1.0)	0	0	0	0	0	0	2
Nebel, Steve^*^	0–0–0	6 (2.0)	0	0	2	1	0	0	0	0
Paas, Fred	0–0–12	8 (1.5)	1 (1.0)	0	4	3	0	0	0	1
Parker, Philip D.	0–6–0	0	19 (1.1)	0	0	2	1	0	0	0
Pekrun, Reinhard	2–1–6	2 (1.0)	17 (1.1)	2	0	3	2	1	0	0
Preckel, Franzis	0–0–7	0	9 (1.0)	0	0	1	0	0	0	0
Rau, Martina A.^*^	3–3–2	5 (1.4)	0	0	3	0	0	0	0	0
Rawson, Katherine A.	0–1–7	9 (2.1)	1 (1.0)	0	1	0	0	0	0	0
Renkl, Alexander	0–1–9	7 (1.6)	0	0	4	0	0	0	1	0
Rey, Günter Daniel	0–1–10	8 (2.2)	0	0	2	2	0	0	0	0
Roberts, Greg	0–0–1	6 (1.0)	3 (1.0)	0	0	0	0	0	0	0
Robinson, Kristy A.^*^	0–4–2	1 (1.0)	9 (1.0)	0	0	0	0	0	0	0
Roelle, Julian	0–7–2	9 (1.7)	0	0	1	0	0	1	0	0
Rosenzweig, Emily Q.^*^	0–5–0	3 (1.0)	3 (1.0)	1	0	0	0	0	0	1
Rutherford, Teomara^*^	2–2–1	0	4 (1.0)	0	0	0	0	0	1	0
Salmela-Aro, Katariina	0–0–8	0	8 (1.0)	0	0	1	0	0	0	0
Scheiter, Katharina	0–1–9	9 (1.2)	2 (1.0)	0	2	0	0	0	0	1
Schneider, Sascha^*^	0–7–2	8 (2.2)	0	0	1	2	0	0	0	0
Schüler, Anne	2–1–0	4 (1.2)	0	0	0	0	0	0	0	0
Schunn, Christian D.	1–0–8	0	7 (1.1)	0	1	0	0	0	0	0
Sinatra, Gale M.	1–1–4	4 (1.2)	2 (1.5)	3	0	0	0	0	0	0
Sweller, John	1–1–11	9 (2.2)	0	1	3	0	0	0	0	0
Trautwein, Ulrich	0–0–14	7 (1.0)	21 (1.1)	0	1	0	1	0	0	3
van Gog, Tamara	0–1–9	13 (1.9)	2 (1.0)	0	3	0	0	0	0	0
Wentzel, Kathryn R.	3–6–0	1 (1.0)	2 (1.0)	0	1	2	0	2	0	0
Wigfield, Allan	0–2–4	1 (1.0)	7 (1.1)	4	0	0	0	0	0	0
Wolff, Fabian^*^	0–9–0	1 (3.0)	5 (1.2)	0	0	1	2	0	0	0
Zhang, Xiao	0–4–2	0	8 (1.0)	0	0	0	0	0	0	0

^*^Early career researchers who received their doctorate in 2013 or later.

^a^Studies such as case studies, interview studies, mixed methods studies, or book reviews.

**Table 6:** Absolute and relative number of different types of articles/studies published by the most productive researchers (*N* = 580).

**Type of article/study**	**Absolute number**	**Percentage**
Correlational study	289	49.83
Experimental study	158	27.24
Review article	42	7.24
Meta-analysis (with or without systematic review)	25	4.31
Theoretical article	23	3.97
Method article	14	2.41
Commentary	12	2.07
Introduction to special issue	11	1.90
Case study or interview study	5	0.86
Book review	1	0.17
Mixed methods study	1	0.17

#### 3.2.2 Number of studies per article

With regard to the studies classified as original studies (i.e., studies with an experimental, correlations, mixed methods, or case study/interview study design), an average of 1.24 studies (*SD* = 0.58) were reported per article. Focusing on the most frequent article types, we observed that the average number of studies per article was slightly but statistically significantly higher (*b* = 0.52, 95% CI [0.44, 0.61]) for studies with an experimental design (*M* = 1.60, *SD* = 0.84) than for studies with a correlational design (*M* = 1.08, *SD* = 0.30).

#### 3.2.3 Topical themes

The results of the keyword analysis are detailed in Table 7. Researchers in the sample published on a wide range of topics within educational psychology. Notably, the most prevalent topics among highly productive researchers (i.e., the top 10 keywords) included motivation (*n* = 24), quantitative methods (*n* = 19), multimedia learning (*n* = 11), achievement (*n* = 9), self-concept (*n* = 9), self-regulated learning (*n* = 9), cognitive load (*n* = 7), emotions (*n* = 6), classroom processes (*n* = 5), and engagement (*n* = 5).

**Table 7:** Overview of most productive researchers' country of institution(s), institutions(s), whether they were identified by the point or count method or were identified by both scoring methods, the year they obtained their PhD, the number of years they have spent in science, and the top 3 keywords to characterize their publications in the present ranking.

**Name**	**Country of institution(s)**	**Institution(s)**	**Top 50**	**Year of PhD**	**Years in science**	**Main PhD supervisor(s)**	**Keyword No. 1 (% of publications)**	**Keyword No. 2 (% of publications)**	**Keyword No. 3 (% of publications)**
Alexander, Patricia A.	USA	University of Maryland, College Park	Point & count	1981	45	Ruth Garner	Knowledge (38%)	Reading (38%)	Relational reasoning (38%)
Bardach, Lisa	Germany	University of Tübingen	Only point	–	–	–	Classroom processes (63%)	Motivation (63%)	Quantitative methods (38%)
Barzilai, Sarit	Israel	University of Haifa	Only point	2012	16	Anat Zohar	Epistemic thinking (75%)	Multiple sources evaluation (75%)	Evaluation strategies (50%)
Becker, Michael	Germany	TU Dortmund University, DIPF | Leibniz Institute for Research and Information in Education	Point & count	2009	18	Oliver Lüdtke	Self-concept (70%)	Big-fish-little-pond effect (50%)	Longitudinal study (50%)
Beege, Maik^*^	Germany	Freiburg University of Education	Only count	2019	8	Günter Daniel Rey	Multimedia learning (50%)	Cognitive load (30%)	Decorative pictures (30%)
Bernacki, Matthew L.	USA	University of North Carolina at Chapel Hill	Point & count	2010	18	James P. Byrnes	Metacognition (33%)	Self-regulated learning (33%)	Digital learning technology (33%)
Bråten, Ivar	Norway	University of Oslo	Only count	1990	35	Ola Bø	Multiple documents (60%)	Epistemic cognition (40%)	Refutation texts (40%)
Burns, Emma C.^*^	Australia	Macquarie University	Point & count	2017	10	Andrew J. Martin	Engagement (60%)	Achievement (60%)	Quantitative methods (30%)
Ching, Boby Ho-Hong^*^	Macau	University of Macau	Only point	2016	10	Terezinha Nunes	Mathematical skills (67%)	Mathematical principles (50%)	Children (50%)
Collie, Rebecca J.^*^	Australia	University of New South Wales	Point & count	2014	12	Jennifer Shapka	Engagement (50%)	Achievement (44%)	Motivation (44%)
Dicke, Theresa^*^	Australia	Australian Catholic University	Only count	2014	13	Detlev Leutner	Motivation (63%)	Big-fish-little-pond effect (31%)	Quantitative methods (31%)
Dumas, Denis^*^	USA	University of Georgia	Only point	2016	13	Patricia A. Alexander	Creativity (33%)	Quantitative methods (33%)	STEM (33%)
Eccles, Jacquelynne S.	USA	University of California, Irvine	Only count	1974	54	Bernard Weiner	Motivation (83%)	Quantitative methods (50%)	Mathematics (33%)
Fiorella, Logan^*^	USA	University of Georgia	Point & count	2015	12	Richard E. Mayer	Multimedia learning (38%)	Generative learning (31%)	Self-regulated learning (15%)
Flanigan, Abraham E.^*^	USA	Georgia Southern University	Only point	2018	11	Kenneth A. Kiewra	Computer science (43%)	Productivity (43%)	Self-regulation (43%)
Fuchs, Lynn S.	USA	Vanderbilt University, American Institutes for Research	Only count	1981	43	Stanley L. Deno	Mathematical skills (89%)	Quantitative methods (89%)	Word problems (56%)
Gaspard, Hanna^*^	Germany	TU Dortmund University	Point & count	2015	11	Benjamin Nagengast, Ulrich Trautwein	Quantitative methods (75%)	Motivation (67%)	Expectancy-value theory (42%)
Geary, David C.	USA	University of Missouri	Point & count	1986	41	Keith Widaman	Mathematical skills (67%)	Development (44%)	Word problems (22%)
Goetz, Thomas	Austria	University of Vienna	Only count	2002	25	Reinhard Pekrun	Emotions (77%)	Quantitative methods (46%)	Motivation (38%)
Göllner, Richard^+^	Germany	University of Tübingen	Only count	–	–	–	Classroom processes (82%)	Quantitative methods (28%)	Motivation (18%)
Graham, Sandra	USA	University of California, Los Angeles	Only point	1982	45	Bernard Weiner	Race/ethnicity (100%)	Diversity (67%)	Mathematics/STEM (33%)
Graham, Steve	USA	Arizona State University	Point & count	1978	48	Floyd Hudson	Writing (86%)	Motivation (43%)	Reading (43%)
Greene, Jeffrey A.	USA	University of North Carolina at Chapel Hill	Point & count	2007	20	Roger Azevedo	Self-regulated learning (25%)	Achievement (17%)	Critical-analytical thinking (17%)
Guay, Frédéric	Canada	Universtité Laval	Point & count	1997	29	Robert J. Vallerand	Motivation (83%)	Self-determination theory (50%)	Quantitative methods (33%)
Guo, Jiesi^*^	Australia	Australian Catholic University	Point & count	2016	10	Herbert W. Marsh	Self-concept (58%)	Big-fish-little-pond effect (26%)	Social comparison (26%)
Hoogerheide, Vincent^*^	Netherlands	Utrecht University	Point & count	2016	11	Sofie Loyens, Tamara van Gog	Generative learning (45%)	Learning by explaining (45%)	Example-based learning (36%)
Jansen, Malte^*^	Germany	Institute for Educational Quality Improvement (IQB)	Only count	2014	12	Ulrich Schroeders	Self-concept (82%)	Longitudinal study (45%)	Quantitative methods (27%)
Kim, Young-Suk Grace	USA	University of California, Irvine	Only point	2007	21	Catherine Snow	Reading (67%)	Language (33%)	Writing (33%)
Klusmann, Uta	Germany	Leibniz Institute for Science and Mathematics Education (IPN), Kiel University	Only count	2008	17	Mareike Kunter, Ulrich Trautwein	Classroom processes (70%)	Emotions (60%)	Social factors (40%)
Köller, Olaf	Germany	Leibniz Institute for Science and Mathematics Education (IPN)	Only count	1997	30	Jürgen Baumert	Motivation (44%)	Comparison processes (33%)	Multimedia learning (33%)
Lachner, Andreas^*^	Germany	University of Tübingen	Point & count	2015	13	Matthias Nückles	Learning by explaining (67%)	Generative learning (56%)	Retrieval practice (22%)
Lauermann, Fani^*^	Germany	TU Dortmund University, University of Bonn	Only point	2013	14	Stuart Karabenick	Motivation (83%)	Teacher characteristics (50%)	Teaching (33%)
Lazarides, Rebecca^*^	Germany	Universität Potsdam, Cluster of Excellence Science of Intelligence (SCIoI)	Only point	2013	14	Angela Ittel	Motivation (88%)	Quantitative methods (75%)	Classroom processes (63%)
Lindner, Marlit Annalena^*^	Germany	Leibniz Institute for Science and Mathematics Education (IPN), Leibniz-Institut für Wissensmedien (IWM)	Only point	2016	12	Olaf Köller	Multimedia learning (63%)	Test-taking motivation (38%)	Cognitive load (25%)
Linnenbrink-Garcia, Lisa	USA	Michigan State University	Only count	2002	26	Paul Pintrich	Motivation (78%)	Achievement (33%)	Engagement (33%)
List, Alexandra^*^	USA	The Pennsylvania State University	Only point	2014	14	Patricia A. Alexander	Multiple texts (80%)	Integration (40%)	Writing (20%)
Lüdtke, Oliver	Germany	Leibniz Institute for Science and Mathematics Education (IPN), Kiel University	Point & count	2004	25	Jürgen Baumert	Quantitative methods (41%)	Self-concept (36%)	Emotions (36%)
Mainhard, Tim	Netherlands	Leiden University	Only count	2009	19	Theo Wubbels, Mieke Brekelmans	Classroom behavior (46%)	Emotions (38%)	Interpersonal behavior (31%)
Marsh, Herbert W.	Australia	Australian Catholic University	Point & count	1972	57	Allen Parducci	Self-concept (55%)	Social comparison (23%)	Big-fish-little-pond effect (19%)
Martin, Andrew J.	Australia	University of New South Wales	Point & count	1999	30	Herbert W. Marsh	Achievement (60%)	Motivation (55%)	Engagement (55%)
Mayer, Richard E.	USA	University of California, Santa Barbara	Point & count	1973	53	James Greeno	Multimedia learning (47%)	Learning strategies (23%)	Virtual reality (17%)
McBride, Catherine	USA	Purdue University	Only count	1994	34	Frank Manis	Linguistic skills (75%)	Literacy (62%)	Family factors (25%)
Möller, Jens	Germany	Kiel University	Point & count	1991	37	Uwe Grau	Dimensional comparison (60%)	Self-concept (60%)	I/E model (52%)
Morin, Alexandre J. S.	Canada	Concordia University	Point & count	2005	23	Michel Janosz, Serge Larivée	Quantitative methods (54%)	Motivation (54%)	Self-determination theory (54%)
Muis, Krista R.	Canada	McGill University	Point & count	2004	22,5	Philip H. Winne	Emotions (64%)	Self-regulated learning (36%)	Elementary students (27%)
Murayama, Kou	Germany	University of Tübingen, University of Reading	Only count	2006	21	Shin-ichi Ichikawa	Motivation (90%)	Quantitative methods (20%)	Reading/literacy (10%)
Nagengast, Benjamin	Germany	University of Tübingen	Only count	2009	18	Rolf Steyer	Motivation (82%)	Achievement (35%)	Quantitative methods (35%)
Nebel, Steve^*^	Germany	University of Potsdam	Only count	2018	10	Günter Daniel Rey	Cognitive load (38%)	Multimedia learning (38%)	Decorative pictures (25%)
Paas, Fred	Netherlands, Australia	Erasmus University Rotterdam, University of Wollongong, University of New South Wales	Only count	1993	34	Jeroen van Merrienboer	Cognitive load (65%)	Self-regulated learning (24%)	Working memory (24%)
Parker, Philip D.	Australia	Australian Catholic University	Point & count	2011	17	Andrew Martin	Self-concept (48%)	Social comparison (29%)	Achievement (19%)
Pekrun, Reinhard	United Kingdom, Australia	University of Essex, Australian Catholic University	Point & count	1982	44	Klaus Schneewind	Emotions (62%)	Achievement (38%)	Control-value theory (35%)
Preckel, Franzis	Germany	University of Trier	Only count	2002	23	Heinz Holling	Quantitative methods (62%)	Self-concept (62%)	Motivation (50%)
Rau, Martina A.^*^	USA, Switzerland	University of Wisconsin-Madison (until 07/31/2023); ETH Zurich (starting 08/01/2023)	Only point	2013	15	Vincent Aleven	Representations (71%)	Sense-making processes (43%)	Drawing (29%)
Rawson, Katherine A.^+^	USA	Kent State University	Point & count	–	–	–	Self-regulated learning (27%)	Example-based learning (27%)	Memory (27%)
Renkl, Alexander	Germany	University of Freiburg	Only count	1991	35	Franz Weinert	Example-based learning (42%)	Learning by explaining (42%)	Self-regulated learning (25%)
Rey, Günter Daniel	Germany	Chemnitz University of Technology	Only count	2007	17	Karl F. Wender	Multimedia learning (60%)	Cognitive load (40%)	Decorative pictures (30%)
Roberts, Greg	USA	The University of Texas at Austin	Only count	1998	30	Toni Falbo	Language learning (67%)	Reading (67%)	Mathematical skills (44%)
Robinson, Kristy A.^*^	Canada	McGill University	Only count	2019	9	Lisa Linnenbrink-Garcia	Motivation (70%)	Science/STEM (60%)	Expectancy-value theory (40%)
Roelle, Julian	Germany	Ruhr University Bochum	Point & count	2011	13	Kirsten Berthold	Self-regulated learning (45%)	Learning processes (45%)	Retrieval practice (27%)
Rosenzweig, Emily Q.^*^	USA	University of Georgia	Only point	2017	11	Allan Wigfield	Expectancy-value theory (100%)	Motivation (100%)	STEM (33%)
Rutherford, Teomara^*^	USA	University of Delaware	Only point	2014	13	Michael E. Martinez, George Farkas	Educational technology (40%)	Metacognition (40%)	Self-regulated learning (40%)
Salmela-Aro, Katariina	Finland	University of Helsinki	Only count	1996	25	Jari-Erik Nurmi	Motivation (71%)	Quantitative methods (43%)	Transitions in educational contexts (29%)
Scheiter, Katharina	Germany	University of Potsdam	Point & count	2003	25	Peter Gerjets	Multimedia learning (42%)	Cognitive load (33%)	Video modeling (25%)
Schneider, Sascha^*^	Switzerland	University of Zurich	Point & count	2017	9	Günter Daniel Rey	Multimedia learning (55%)	Cognitive load (45%)	Decorative pictures (27%)
Schüler, Anne	Germany	Leibniz-Institut für Wissensmedien (IWM)	Only point	2010	17	Katharina Scheiter	Multimedia learning (100%)	Eye tracking (75%)	Integration processes (75%)
Schunn, Christian D.	USA	University of Pittsburgh	Point & count	1995	33	David Klahr	Engagement (33%)	Science (33%)	Feedback (22%)
Sinatra, Gale M.	USA	University of Southern California	Point & count	1989	35	James Michael Royer	Conceptual change (57%)	Text processing and learning (57%)	Refutation texts (43%)
Sweller, John	Australia	University of New South Wales (Professor Emeritus)	Point & count	1972	51	Tony Winefield	Cognitive load theory (79%)	Element interactivity (21%)	Expertise reversal effect (21%)
Trautwein, Ulrich	Germany	University of Tübingen	Point & count	2002	24	Olaf Köller	Motivation (43%)	Classroom processes (28%)	Achievement (20%)
van Gog, Tamara	Netherlands, Germany	Utrecht University, University of Tübingen (Distinguished International Professor)	Only count	2006	21	Fred Paas, Jeroen van Merriënboer	Example-based learning (33%)	Multimedia learning (22%)	Learning by teaching (22%)
Wentzel, Kathryn R.	USA	University of Maryland, College Park (Professor Emerita)	Point & count	1987	40	Martin Ford	Social factors (100%)	Motivation (67%)	Quantitative methods (67%)
Wigfield, Allan	USA, Germany	University of Maryland, College Park (Professor Emeritus), University of Heidelberg (Honorary Professor)	Point & count	1982	45	Steven R. Asher, Kennedy T. Hill	Motivation (90%)	Expectancy-value theory (50%)	Dimensional comparison (30%)
Wolff, Fabian^*^	Germany	University of Koblenz	Point & count	2018	7	Jens Möller	Dimensional comparisons (100%)	I/E model (100%)	Self-concept (89%)
Zhang, Xiao	Hong Kong	The University of Hong Kong	Only point	2009	17	Shui-fong Lam	Mathematics (75%)	Language/reading (50%)	Self-regulation (25%)

^*^Early career researchers who received their doctorate in 2013 or later.

+ Information obtained via Google Scholar, ORCID, ResearchGate, and/or university websites.

– Information not available.

#### 3.2.4 Collaboration

On average, unique publications published by the most productive researchers had 4.20 authors (*SD* = 1.80), and the highest number of authors was 11. Only approximately 5% of the publications had a single author. Thus, approximately 95% of the publications had two or more authors. The percentage of first author positions was about 23% and of last author positions 32%. Because the importance of the last author position varies from publication to publication and can be controversial, we surveyed the views of the most productive researchers on the role of the last author in a scientific publication. The greatest percentage of researchers (35%) consider the last author's position to be the second most important role, surpassed only by the first author. In contrast, 27% consider it to be the least important, and an equal number (16%) either consider it to be of varying importance or consider it to be the position of the senior author of the project. Taken together, there is no consensus on the role of the last author position; its importance is likely to vary with context (for details, see Supplementary Section S2.1 in the OS).

### 3.3 Supervision of PhD students and PostDocs

Highly productive researchers (*n* = 70) reported to have supervised, on average, 17.37 PhD students (*SD* = 14.45, range: 0–60), and 5.60 PostDocs (*SD* = 7.32, range: 0 and 40) during their research career. Information regarding the main supervisor(s) and inspiring role model(s) of the highly productive researchers are provided in the OS (Supplementary Tables S2.2, S2.3).

### 3.4 Open science practices

Open science practices were rarely used by the most productive researchers. None of their studies were registered reports and only 1.38% (*n* = 8) were preregistered. Among the most productive researchers, nine had authored preregistered articles. The first preregistered study in our sample was published in 2020. There were four preregistered studies published in 2021 and three in 2022. Two preregistered studies were published in each of the journals *Contemporary Educational Psychology, Educational Psychology Review, Journal of Educational Psychology*, and *Learning and Instruction*. However, in *Educational Psychologist* and *Journal of the Learning Sciences*, there were no preregistered studies published.

### 3.5 Diversity in the group of highly productive researchers in educational psychology

#### 3.5.1 Diversity in sample characteristics

##### 3.5.1.1 Age

The mean age of respondents in 2023 was 50.57 years (*SD* = 13.53, *n* = 69) with a range of 32–79 years, indicating substantial variation in age within this group. On average, the most productive researchers had spent 23.81 years (*SD* = 13.21, *n* = 71) in science, including their PhD time, ranging from 7 to 57 years in 2023.

##### 3.5.1.2 Gender

Of 71 respondents, 39% reported being female, 1% non-binary, and 59% male. Thus, women are underrepresented in the group of highly productive researchers.

##### 3.5.1.3 Race/ethnicity

With regards to the racial and ethnic origins of the group of highly productive researchers, 86% self-identified as White, 7% as Asian, 1% as Black, 3% preferred to self-identify (Jewish), and 3% preferred not to answer (*n* = 70). None of the highly productive researchers self-identified as Latinx, Middle Eastern, or Mixed Race. Of those who self-identified as Asian, all self-identified as East Asian (e.g., Chinese, Japanese, Korean); of those who self-identified as Black, all self-identified as North American (e.g., Canadian, American); and of those who self-identified as White, 75% self-identified as European (e.g., British, French, Polish, Russian), 18% as North American (e.g., Canadian), 5% as European and North American, and 2% self-identified as European Jewish.

##### 3.5.1.4 Nationality

The largest percentage of highly productive researchers are of German nationality (37%), followed by US nationality (34%), and Australian nationality (7%). Researchers of Canadian, Chinese, and Dutch nationality account each for 4%, and the remaining 5% are of various nationalities (Bulgarian, Canadian, Finnish, Israeli, Japanese, and Norwegian). Notably, when applying the point-based method, researchers from the United States occupy most of the top-ranked positions (7 out of 10). In contrast, when using the count-based or combined method, the top 10 include a more balanced representation of researchers with U.S., German, and Australian nationalities.

##### 3.5.1.5 Continents and countries

**Figure 1:**
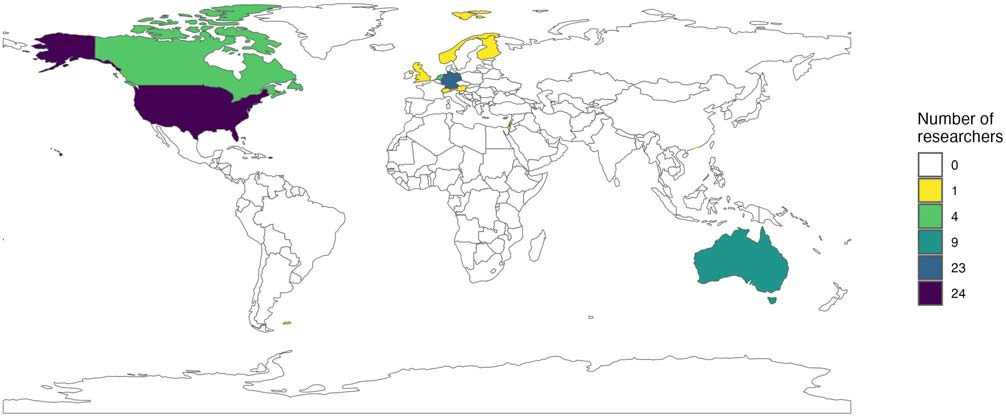
World map of countries where the identified most productive researchers are employed.

As illustrated in Figure 1, the majority of the most productive researchers (*n* = 71) worked in Europe (*n* = 32; 45%: *n*_Germany_ = 23; *n*_Netherlands_ = 4; *n*_Austria_ = 1; *n*_Finland_ = 1; *n*_Norway_ = 1; *n*_Switzerland_ = 1; *n*_UK_ = 1), North America (*n* = 28; 40%: *n*_*US*_ = 24; *n*_*Canada*_ = 4), and Australia (*n* = 9; 13%), but were considerably less employed in other parts of the world (*n* = 3; 4%; *n*_HongKong_ = 1; *n*_Israel_ = 1; *n*_Macau_ = 1)^2^. Thus, 67% of the most productive researchers were employed at non-US institutions, reflecting an increasing internationalization of highly productive researchers over the years, which has also been shown in prior ranking studies (1991–2002: 16%; 2003–2008: 50%; 2015–2021: 46% at non-US institutions).

##### 3.5.1.6 Institutions

Highly productive researchers were most frequently employed^3^ at the University of Tübingen (Germany; *n* = 7) and the Australian Catholic University (Australia, *n* = 5), followed by the Leibniz Institute for Science and Mathematics Education (IPN; Germany) and the University of New South Wales (Australia; both *n* = 4). Three researchers each were affiliated with Kiel University (Germany), the Technical University of Dortmund (Germany), the University of Georgia (US), the University of Maryland, College Park (US), and the University of Potsdam (Germany). For a complete list of institutions, see Supplementary Table S2.4 in the OS; for individual researchers' institutional affiliations, see Table 7.

##### 3.5.1.7 Flexibility of location

**Figure 2:**
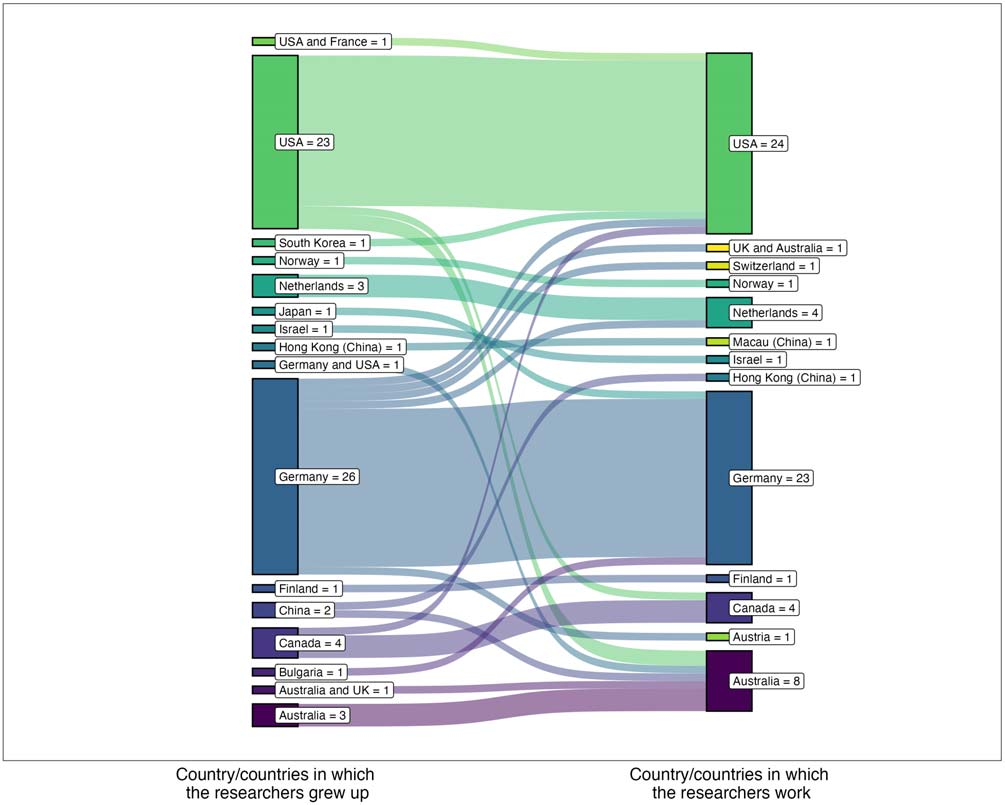
Movement between most productive researchers' native countries and current countries of employment.

While most highly productive researchers had moved within a country at least once for a job (79%), a significant majority (72%) had not moved to another country for a job (Table 8). Figure 2 provides a detailed picture of migration patterns between researchers' countries of origin and their current affiliations. The majority of researchers remain employed in their country of origin. In particular, Australia emerges as a significant attractor of highly productive researchers from abroad, while Germany shows a tendency for researchers to move to other countries. Australia retains all of its highly productive researchers and in particular gains four from China, Germany, and the United States. In contrast, Germany experiences a net loss, losing five researchers to Australia/the United Kingdom, Austria, the Netherlands, Switzerland, and the United States, while gaining only two researchers from Japan and Bulgaria. The United States maintains a balance, losing as many highly productive researchers to countries like Australia and Canada as it gains from countries like South Korea, Germany, and Canada.

**Table 8:** Flexibility in terms of moving within and across countries for a job of the most productive researchers in educational psychology journals from 2017 to 2022.

**Number of moves**	**% of moves within a country**	**% of moves across countries**
0	21	72
1	34	13
2	20	11
3	17	1
4	4	0
5	3	1
6	1	1

##### 3.5.1.8 Language

Fifty-nine percent indicated that English was not their first language (*n* = 70). Of those researchers whose first language was not English, 7% moved to an English-speaking country while growing up at the ages of 4, 5, or 23 (*n* = 42).

##### 3.5.1.9 First-generation student and faculty status

In the context of researchers' educational backgrounds and faculty affiliations, the data revealed interesting patterns. Of the 71 respondents, 39% reported that none of their primary caregivers, such as biological parents, had a university degree. Conversely, 35% reported that one of their primary caregivers had a university degree, while 25% reported that multiple primary caregivers in their family had such a degree.

Looking at the educational attainment of the previous generation (i.e., the researchers' grandparents), 73% reported that none of their primary caregivers' primary caregivers (hereafter referred to as “grandparents”) had a university degree. Meanwhile, 18% reported having one grandparent with a university degree, and 8% reported having several grandparents with such academic credentials.

In terms of faculty affiliation, the majority (86%) reported that their primary caregivers had never held a faculty position. In contrast, 13% had a primary caregiver who had held a faculty position. Only 1% of respondents reported having multiple caregivers who had held faculty positions. Within this subset of highly productive researchers, 97% reported that none of their grandparents had ever held a faculty position. Within the same group, only 1% reported having one or more grandparents with faculty affiliations.

#### 3.5.2 Diversity in positions

Examining the academic positions held by highly productive researchers, about 93% had some form of professorial status. The majority (56%) were full professors (Table 9), and about 7% held positions as PostDocs, research scientists, or senior lecturers in 2023.

**Table 9:** Academic positions of the most productive researchers in educational psychology journals from 2017 to 2022.

**Position**	**Total percentage (*n* = 71)**	**Percentage in the group of women (*n* = 28)**	**Percentage in the group of men (*n* = 42)**
PostDoc	3	4	2
Research scientist	1	0	2
Senior lecturer	3	4	0
Junior professor	1	0	2
Assistant professor	10	7	12
Associate professor	18	25	14
Full professor (tenured)	56	50	62
Full professor (without tenure, 6-year contract)	1	4	0
Distinguished professor	1	4	0
Emeritus/emerita professor	4	4	5

### 3.6 Activities related to professional, housework, care, nursing, or custodial work

The average weekly hours spent in a typical term on work, teaching, housework, as well as care, nursing, or custodial work are shown in Table 10.

**Table 10:** Weekly hours spent (mean and standard deviation) on work-related activities and caregiving, nursing, or custodial work.

**Activities**	***M* (*SD*), Range**	**Women**	**Men**	**Difference**
**Weekly hours on average (typical term)**				
Working hours (*n* = 71)	48.60 (9.33), 30–70	48.07 (9.66)	48.56 (8.96)	ns
Teaching activities (*n* = 71)	6.63 (7.63), 0–50	6.02 (3.36)	6.67 (8.89)	ns
Housework (*n* = 70)	10.27 (6.59), 1–35	10.09 (7.21)	9.86 (6.30)	ns
Care, nursing, or custodial work (*n* = 45)	26.96 (21.33), 2–128	35 (27.14)	20.52 (12.42)	^*^
**Spending working hours in %**				
Research activities (*n* = 71)	48.60 (9.33), 10–80	42.57 (14.71)	39.76 (19.63)	ns
Teaching activities (*n* = 62)	20.86 (11.69), 0–60	20.30 (9.61)	20.75 (12.98)	ns
Supervision activities (*n* = 66)	19.16 (9.47), 2–45	20.20 (8.23)	18.86 (10.10)	ns
Administrative activities (*n* = 62)	17.89 (14.16), 0–80	16.08 (10.95)	19.08 (16.22)	ns
Other activities (*n* = 37)	9.49 (7.55), 0–40	10.23 (5.46)	9.26 (8.70)	ns

In terms of the distribution of working hours, highly productive researchers dedicated 40.73% (*SD* = 17.68) of their work hours to research (*n* = 71), 20.86% (*SD* = 11.69) to teaching (*n* = 62), 19.16% (*SD* = 9.47) to supervision (*n* = 66), and 17.89% (*SD* = 14.16) to administrative tasks (*n* = 62) during a typical term. The rest of their working hours (9.49%, *SD* = 7.55) was spent on other tasks.

Furthermore, among highly productive researchers, 70% confirmed their involvement in some form of care work at various points in their careers, while 28% reported no such involvement (*n* = 71). At the time the survey was administered, 39% of highly productive researchers are engaged in care work, while 31% have done so in the past. In this group of scholars, 68% reported having at least one child (*n* = 69) and providing an average of 12.92 years (*SD* = 7.37) of care, nursing, or custodial work during their academic careers (*n* = 45). Highly productive researchers managed, on average, 47.92% (*SD* = 19.66) of their care responsibilities, indicating that the remainder was shared with others (*n* = 50).

### 3.7 Gender differences related to professional, housework, care, nursing, or custodial work

As can be seen in Table 10, there were no gender differences in the group of highly productive researchers regarding the number of working hours, the amount of teaching, and housework. Furthermore, both genders allocated similar amounts of time to research, teaching, supervision, administrative, or other activities, and appear to be comparable in terms of child rearing and care during their careers [women: 82%; men: 66%; χ^2^_(1)_ = 1.471, *p* = 0.225]^4^. Additionally, women were as likely as men to have children (*b* = −0.02; 95% CI [−0.48, 0.43]). Among those highly productive researchers who have provided care work in the past, women (14.00 years, *SD* = 7.68) reported a similar amount of caregiving as men (17.85 years, *SD* = 8.32; 95% CI [−3.45, 11.14]). However, women overall reported to take a significantly greater share of care responsibilities per week (*M* = 58.48%, *SD* = 20.70) than men (*M* = 38.93%, *SD* = 13.52%; *b* = −14.48, 95% CI [−26.75, −2.21]), and reported significantly higher hourly commitments to care, nursing, or custodial work during their academic careers (*M* = 35.00 h, *SD* = 27.14) than men did (*M* = 20.52 h, *SD* = 12.42; *b* = −14.48, 95% CI [−26.75, −2.21]).

## 4 Discussion

In the present study, we examined the individual productivity of highly productive researchers in educational psychology by analyzing articles published in top-tier, broad-scope educational psychology journals between 2017 and 2022. We aimed to update and extend prior ranking studies and to address limitations and blind spots of prior research. To this end, we used an objective criterion to select top-tier and broad-scope educational psychology journals based on the average rank of the journals in the “Psychology, Educational” list of the Journal Citation Reports from 2017 to 2022, capitalized on more recent publication data, applied three scoring methods, set a larger target number of highly productive researchers (i.e., the top 50), and conducted an online survey to shed light on their characteristics (e.g., demographics, working habits, and care responsibilities). We also examined publication trends in terms of research topics, open science practices, collaboration, and internationalization of research.

### 4.1 Individual productivity

Overall, the count-based, point-based, and combined methods resulted in different lists, with a substantial overlap in the researchers listed (i.e., 45% of the researchers were identified by all three scoring methods, 23% were identified by only two scoring methods, and 32% were identified by only one scoring method). Variations between these lists might reflect that highly productive researchers have different publication strategies and/or different roles in collaborative projects. One such strategy involves taking on more supervisory roles in research teams and thus occupying the last author position, which is penalized in the point-based scoring method but not in the count-based method. For example, based on the point-based (but not count-based) scoring method, Patricia A. Alexander (#2) and Logan Fiorella (#7) were ranked among the 10 most productive researchers, suggesting they are less likely to occupy the last author position in their publications. In contrast, based on the count-based (but not the point-based) scoring method, Ulrich Trautwein (#2), Oliver Luüdtke (#6), and Tamara van Gog (#10) appeared among the top 10 (for the full lists of highly productive researchers based on each scoring method, see Tables 2–4).

As in prior ranking studies, senior researchers achieved the highest ranks. Specifically, from 2017 to 2022, three researchers (i.e., Richard E. Mayer, Reinhard Pekrun, and Herbert W. Marsh) appeared in the top 5 across all three rankings. Notably, Richard E. Mayer and Herbert W. Marsh have consistently been listed among the top-performing researchers since 1991 (Fong et al., 2022; Greenbaum et al., 2016; Hsieh et al., 2004; Jones et al., 2010; Smith et al., 1998, 2003). Using the point-based ranking method, Patricia A. Alexander has additionally been listed among the top-performing researchers since 1991, and for the count-based method John Sweller has additionally been included. Alongside established researchers, the rankings also shed light on the rise of early career researchers. Notably, 40% of the top 50 in the point-based ranking were early career researchers, whereas 25% of the individuals in the count-based ranking were early career researchers, and 37% in the combined ranking (e.g., Rebecca J. Collie, Logan Fiorella, Hanna Gaspard, and Vincent Hoogerheide appeared in all three rankings). These statistics provide a glimpse into the diversity of contributors to the field, including both seasoned and emerging researchers.

### 4.2 Publication trends

We observed several important publication trends. First, multiple-study publications are still rare, even in the selected top-tier journals in educational psychology. In this analysis, an average of only 1.24 studies was reported per article. Future studies are encouraged to include more than one study per publication to strengthen the evidence and replicability of findings (for a similar argument, see Plucker and Makel, 2021).

Second, most of the studies published in the selected journals by the most productive researchers were quantitative in nature. Most articles had a correlational study design (50%) or an experimental study design (27%), while less than 1% of the studies involved qualitative research (e.g., interview study designs or mixed methods study designs). This finding speaks to the trend in educational psychology toward a greater percentage of observational and correlational studies (Brady et al., 2023; Hsieh et al., 2004), with a decrease in the percentage of experimental studies (Brady et al., 2023).

Third, we observed a persistent trend toward increased collaboration. In the present ranking study, most productive researchers published only 5% of their articles as single authors, while a substantial majority of 95% were collaborations, with an average of 4.20 authors per publication. In prior ranking studies, publications involved fewer authors (see e.g., Fong et al., 2022; Jones et al., 2010). These results also show that, overall, the most productive researchers are “collaborative researchers.”

Fourth, our ranking study is the first to shed light on the adoption of open science practices by assessing the number of preregistered articles and registered reports published by the most productive researchers. We found that only 1.38% of the articles were preregistered, while none of the studies qualified as a registered report. We expect to see more preregistrations in the future, as indicated by the slight increase in preregistrations between 2017 and 2022—likely also due to the increased adoption of open science policies by many journals in recent years (e.g., in the *Journal of Educational Psychology, Learning and Instruction*). To help our research to become more open, transparent, reproducible, and replicable, future ranking studies are encouraged to continue to assess the extent to which the most productive researchers in educational psychology preregister their hypotheses, sample sizes, and analysis plans. Moreover, we encourage future ranking studies to extend our efforts by considering a wider range of open access practices, such as whether researchers openly share their data, materials, and/or (reproducible) code.

Finally, the research topics of highly productive researchers were very diverse. Our analysis of the keywords used in the publications revealed that the 10 most frequently listed topics (among the top 3 keywords used for publication) were: motivation, quantitative methods, multimedia learning, achievement, self-concept, self-regulated learning, cognitive load, emotions, classroom processes, and engagement. All of these can be considered classic educational psychology topics. While contemporary topics such as digital learning technology and virtual reality appeared once, other emerging trends such as artificial intelligence and personalized learning have not yet become prominent keywords among the most productive researchers in publications from 2017 to 2022. However, we expect that these emerging topics are likely to gain prominence alongside established classical topics in future publications in top-tier, broad-scope educational psychology journals.

### 4.3 Working habits

On average, highly productive researchers reported working 48.60 h and teaching 6.63 h per week in a typical term. It is noteworthy that they reported spending the largest proportion of their working time (about 41%) on research-related activities, followed at some distance by teaching-related activities, supervision, administrative activities, and other activities. The time spent focusing on research activities is certainly a pivotal factor for individual productivity.

### 4.4 Diversity of sample characteristics

A key contribution of our study is that it sheds light on the diversity and international involvement of highly productive researchers, as these factors are beneficial to the scientific advancement of educational psychology. Our results showed that most of the highly productive researchers self-identified as White (86%) and male (59%), did not have a first-generation student status (60%) but did have a first-generation faculty status (86%), did not speak English as their first language (59%), and have moved within countries for their career (79%) but not across countries (72%). Overall, diversity was limited, particularly with regard to race/ethnicity but also gender.

With respect to gender, women and men within the group of highly productive researchers reported broadly similar behaviors in terms of housework and/or care work, childbearing, and work habits. However, somewhat fewer women than men reach this level of productivity, and highly productive female researchers report more hours engaged in care work per week and a higher share of care work within their family. The underrepresentation of women among highly productive researchers may be linked to the attrition of women at the postdoctoral stage—a period that often coincides with the onset of family responsibilities, making it challenging to manage career uncertainties alongside caring responsibilities. In addition, women's greater caring responsibilities, also observed in the present study, may constrain available research time and thereby reduce productivity (e.g., Ysseldyk et al., 2019).

The underrepresentation of certain racial and ethnic groups among the most productive researchers likely reflects multiple structural factors, including systemic barriers such as historical and ongoing institutional bias and discrimination that create barriers to education and career opportunities, but also a lack of representation and role models in academia (e.g., Dupree and Boykin, 2021; Dupree and Kraus, 2022; Fox Tree and Vaid, 2022). In addition, research in the US context indicates that members of minority groups in academia often carry a heavier service load, including committee work, mentoring, and community engagement (Fox Tree and Vaid, 2022; Syed, 2017), which can reduce time available for research and publication.

Our results also showed substantial global imbalances. Most highly productive researchers had European (45%), North American (39%), or Australian (7%) nationalities, whereas only a minority had Asian nationalities (6%), and none were African or South American citizens. Similarly, most respondents were employed in Northwestern Europe (45%), the United States (34%), Australia (13%), or Canada (6%). These disparities are likely linked to structural inequalities across countries and continents. High-income countries typically invest substantially more in research and development, providing better funding structures, infrastructures, and opportunities for scientific careers. For example, high-income countries spend 2.76% of their GDP on research and development, compared to 1.25% in middle-income and 0.21% in low-income countries [United Nations Educational, Scientific and Cultural Organization (UNESCO), 2023]. Researchers from some countries also face visa restrictions for international conferences, research stays, or relocation to other countries, which can hinder career progression. In addition, locally concentrated clusters of highly productive researchers may reflect training lineages: Highly productive researchers may pass on effective research practices to their doctoral students and postdoctoral researchers, increasing the likelihood that these individuals also become highly productive. We saw this in the advisor/advisee relationships in the top 50 most productive researchers (see below and Supplementary Table S.2.2 of the OS), and this assumption is also supported by expertise research (e.g., Ericsson, 2009).

A central question arising from these findings is why diversity within the research community matters in the first place. Prior research shows that diverse research communities generate more innovative and higher-impact science. Diverse teams produce publications with more citations and higher peer-rated quality (AlShebli et al., 2018; Bosetti et al., 2012; Campbell et al., 2013), and researchers from underrepresented groups contribute disproportionately to scientific novelty (Hofstra et al., 2020). Diversity also improves the epistemic process by broadening research questions, revealing biases in existing models, and enabling more accurate data collection—particularly in studies involving human participants (Intemann, 2009). Beyond demographic aspects, global diversity similarly broadens the epistemic scope of psychological research and strengthens generalizability across sociocultural contexts (Lin and Li, 2023b). Thus, the demographic patterns observed in our sample may indicate missed epistemic opportunities. Promoting diversity within educational psychology is therefore not only a matter of representation but directly relevant to scientific quality and innovation.

To increase diversity in educational psychology, Kucirkova (2023) recommends providing diverse mentors who can help students and early career researchers from marginalized groups navigate the implicit structures and routines of academia (i.e., the “hidden curriculum”). Moreover, strengthening dispositions such as confidence and self-efficacy—often lower in women yet crucial for leadership positions—in professional development is important (Howe-Walsh and Turnbull, 2016). Addressing the extreme work culture in academia, which disproportionately affects women and minorities, could additionally be effective, for example, through training in workload management and wellbeing strategies (Kucirkova, 2023). Universities should cultivate environments that support individuals, especially those from marginalized groups, through failures and setbacks and recognize the diverse experiences and challenges one faces in academic careers (Kucirkova, 2023). Further recommendations include increasing diversity among editors, reviewers, authors, and participants, and introducing recognition systems (e.g., badges) for publications that involve research teams or samples extending beyond White, Educated, Industrialized, Rich, and Democratic (WEIRD) populations (Lin and Li, 2023a,b; Roberts et al., 2020). Several initiatives within educational psychology indicate growing attention to diversity—for example reduced conference fees for researchers from low-GDP countries, childcare services at major conferences, awards recognizing minority scholars, lower open-access fees for scholars from low-income countries, and recent racial reckoning efforts across professional organizations [e.g., American Educational Research Association (AERA), 2020; American Psychological Association (APA), 2021]. These measures represent important first steps, yet more sustained and comprehensive efforts will be necessary to address ongoing structural inequities in the field.

### 4.5 Strengths, limitations, and directions for future research

Relative to prior research, we have identified a more extensive list of the most productive researchers (i.e., the top 50) and applied more objective criteria for selecting the top-tier, broad-scope educational psychology journals, increasing the number of target journals from five to six. We focused on publications in these journals because publication volume in top-tier outlets remains a common proxy for research productivity (e.g., Smith et al., 1998) and captures aspects of both quantity and quality (Prinz et al., 2020). Acknowledging that this proxy is reductionistic and does not fully capture the breadth of scientific contributions (Griffin et al., 2023; Schönbrodt et al., 2025), we used both count- and point-based scoring methods and a combined scoring method, and assessed additional indicators of research quality (e.g., number of studies per article, adoption of open science practices). While these enhancements strengthen the present study, several limitations remain and point to directions for future research.

First, although we have extended the list of most productive researchers to 50, future research may benefit from identifying an even higher number of highly productive researchers (e.g., top 500). This would also allow for more diversity in the sample and thereby more fine-grained intersectional analyses (e.g., how different intersectional groups, such as women of color, are represented among highly productive researchers) that may also consider the role of highly productive researchers' socioeconomic status (e.g., parental educational level, income) and its association with researchers' academic career.

Second, while we have considered the authorship position of the most productive researchers in educational psychology, future research may examine more explicitly the role and distributed influence of the collaborative network of co-authors and specify their contributor roles using the metadata of the CRediT taxonomy, at least for more recent publications.

Third, in contrast with prior ranking studies, we focused on top-tier, broad-scope journals within educational psychology, using an objective inclusion criterion of an average rank of ≤ 10 on the Web of Science “Psychology, Educational” list (2017–2022). This threshold expanded the set of journals included in earlier studies, while still restricting the selection to outlets that can reasonably be considered top-tier. Future research may conduct sensitivity analyses to examine potential benchmark effects by varying this threshold (e.g., ≤ 5 or ≤ 15) or the publication window (e.g., 5 vs. 30 years). Furthermore, we excluded both specialized high-impact journals within educational psychology (e.g., *Child Development, Journal of School Psychology, Journal of Counseling Psychology*) and broader high-impact journals outside the scope of educational psychology (e.g., *Educational Research Review, Psychological Bulletin*). This decision aligns with the rationale underpinning prior ranking studies: journals that cover the breadth of educational psychology and are not tied to specific subfields or populations offer a more balanced representation of the field than specialized outlets that may disproportionately reflect larger or more active subdomains. However, limiting the analysis to top-tier, broad-scope educational psychology journals may overemphasize productivity within this specific set while overlooking interdisciplinary work published in broader psychology or education journals, as well as high-quality research on specialized topics typically appearing in niche outlets. To capture a fuller range of productivity profiles within and across educational psychology, future research could incorporate both broad-scope journals beyond the educational psychology category and high-impact specialized outlets. Such an expanded scope would not only increase the likelihood of identifying highly productive researchers who publish across diverse contexts but also enable examinations of the external validity of prior ranking results—specifically, the extent to which observed productivity patterns generalize to educational psychology in its broadest sense.

Fourth, future ranking studies should continue to incorporate and emphasize indicators of research quality (e.g., Vazire and Holcombe, 2022). We recommend expanding the evaluation of high-quality science to include the rigorous application of appropriate research methods (e.g., state-of-the art statistical techniques, representative sampling, design-informed power analyses) and open science practices (e.g., preregistration, registered reports, embedded replication studies, and the sharing of data, materials, and reproducible analysis scripts). These indicators could be assessed using an explicit and transparent scoring scheme for research quality (Leising et al., 2022), thereby helping to recognize and incentivize high-quality, impactful, and reliable research. In addition, preregistering future ranking studies themselves—including the operationalization of productivity and the planned scoring procedures—would further strengthen transparency and methodological rigor.

Fifth, it is debatable whether existing ranking studies—including the present one—adequately capture the full breadth of what a “productive researcher” should be and accomplish. Work to date has relied on publication volume in selected journals as a proxy for productivity. We recommend expanding the conceptualization of research productivity to reflect not only the frequency of publication but also the quality, influence, and broader contributions of researchers. Ideally, a productive researcher would regularly publish high-quality work that also has a meaningful and lasting impact. Such impact may be reflected in high citation counts within and outside the field, which has been recently operationalized by field-specific and field-across impact indices of researchers by factoring in the number of citations within and/or across fields (see Wu et al., 2026). Beyond publication count and impact, productivity may also involve translating research into authentic educational settings, generating influential theoretical contributions, and contributing to the scientific ecosystem—for example, by securing external funding, mentoring junior scholars, and building research infrastructures. Active involvement in editorial and administrative roles (e.g., serving as an associate or chief editor, reviewing for journals, organizing conferences) further demonstrates engagement in advancing the field. Incorporating these additional factors would allow future evaluations to more comprehensively capture the multifaceted nature of scientific productivity, though an operational and transparent scoring framework to assess these contributions has yet to be developed. Broadening the definition of productivity may ultimately help incentivize and reward high-quality, high-impact, and high-engagement scholarship.

Sixth, the most productive researchers in our sample were predominantly based in Northwestern Europe and the United States, likely because universities in these regions provide particularly favorable conditions for research (e.g., strong funding structures, high-quality education, and advanced research infrastructures). Understanding who becomes highly productive—and which groups remain underrepresented—can help the field reflect on potential structural barriers and consider how greater diversity may strengthen the epistemic breadth and societal relevance of educational psychology. Although educational psychology operates within a global scientific community, research systems, resources, and publication practices vary substantially across regions. To examine whether the conclusions drawn in the present study also hold in other contexts, future work could conduct analogous ranking studies and surveys within specific continents, countries, or regions with lower publication density, such as Latin America or Africa. Such locally grounded analyses would help determine the extent to which our findings generalize beyond high-income research environments, identify visibility or language-related biases, and shed light on region-specific structural barriers. They may also inform targeted capacity-building efforts for local researchers and institutions and contribute to a more globally inclusive understanding of research productivity in educational psychology. Follow-up interview studies could further identify factors and strategies that enable researchers in diverse global contexts to become highly productive relative to their peers, and shed light on conditions that promote productivity among specific social groups (e.g., by race/ethnicity, socioeconomic status, gender) that are currently underrepresented in productivity rankings (see also Kiewra and Creswell, 2000; Prinz et al., 2020).

Addressing these research avenues would further enhance and complement the assessment of researchers' productivity within educational psychology.

### 4.6 Implications for (future) early career researchers

Identifying highly productive researchers and understanding the factors associated with high productivity is valuable to current and future generations of researchers. Research productivity continues to be a key criterion for tenure, merit awards, and research funding (Győrffy et al., 2020). Although the present study cannot make causal claims about why the top 50 researchers are highly productive, students and early career researchers can learn from the environments in which these researchers work as well as from their characteristics, work habits, and publication strategies. Insights from ranking studies may therefore serve as one criterion when choosing a supervisor or institution and when planning an academic career.

One plausible hypothesis is that early career researchers may be more likely to become highly productive when mentored by highly productive scholars. This possibly is reflected in several mentor–mentee pairs in our sample [e.g., Richard Mayer (mentor)—Logan Fiorella (mentee); Tamara van Gog (mentor)—Vincent Hoogerheide (mentee); Herbert W. Marsh (mentor)—Jiesi Guo (mentee)]. As discussed earlier, research productivity encompasses not only publication volume and impact but also engagement within the educational psychology community. Adopting this broader understanding may help encourage early career researchers to pursue high-quality, high-impact, and high-engagement scholarship.

Many top-performing researchers emphasize that influence ultimately matters more than sheer output. As Patricia Alexander put it, “Do not aim to be a prolific scholar; aim to be the best and most influential scholar you can be” (p. 39; Patterson-Hazley and Kiewra, 2013). Accordingly, junior researchers should strive to conduct high-quality, impactful research and contribute to the educational psychology community (for further advice, see also Kiewra et al., 2021; Kiewra, 2025; Prinz et al., 2020).

## 5 Conclusions

The present ranking study extends prior work by applying a more objective journal selection and multiple scoring methods to identify the 50 most productive researchers in educational psychology. In addition, we conducted an online survey to examine key characteristics of these researchers and analyzed their publications with respect to research topics, open science practices, collaboration patterns, and internationalization. Our findings shed light on the demographic composition of highly productive researchers, highlighting diversity patterns that may bear on the epistemic breadth and inclusiveness of the field. We additionally outlined directions for future ranking studies, discussed implications for current and future early career researchers, and reflected on how the conceptualization of research productivity might be broadened. Taken together, the present study provides a methodological framework for future productivity research and offers insights that may help early career researchers better understand the conditions and characteristics associated with high research productivity in educational psychology.

## Author's note

When the manuscript for this article was drafted, all authors self-identified as White European. Three authors identified as female and three authors self-identified as male. One author has a first-generation student status, whereas five of the authors' parents or grandparents had a university degree. None of the authors' parents or grandparents had a faculty position. One author was of Dutch nationality, one author was of German and Swedish nationality, and four authors were of German nationality. None of the authors speak English as a first language, nor did they grow up bilingual with English as one of their main languages. At the time, two authors were students, two authors were PostDocs, and two authors had faculty positions.

## Data Availability

The raw data supporting the conclusions of this article will be made available by the authors, without undue reservation, for the journal-based data.

## Footnotes

1 When the number of researchers with the same number of publications exceeded the available top 50 slots in the count-based scoring method, we expanded the top 50 to include everyone with that number of publications.

2 One individual indicated to be employed in two countries, thus we counted these countries separately.

3 If researchers were already retired, they reported the university/institution where they were last employed.

4 It could be argued that our sample of highly productive researchers in educational psychology represents the population, and thus the use of inferential statistics is not necessary. However, we consider the use of inferential statistics appropriate for two reasons. First, our sample is not complete because three individuals did not respond to our survey. Second, we think that the underlying theoretical population is larger than the surveyed researchers because it includes, for example, future highly productive researchers (Gelman, 2009; Krieger, 2012).
