# Supplementary material for: Identifying the 50 most productive researchers in top-tier, broad-scope educational psychology journals (2017–2022): a new perspective with a focus on publication trends and diversity
Source: Front Psychol. 2026 Mar 2;16:1660783. doi: 10.3389/fpsyg.2025.1660783 (PMC12989519; doi:10.3389/fpsyg.2025.1660783)
Supplement: Supplementary file 1 [file Data_Sheet_1.docx]

Supplementary Materials

**Identifying the 50 most productive researchers in top-tier, broad-scope educational psychology journals (2017–2022): A new perspective with a focus on publication trends and diversity**

**Overview**

S1: Supplemental Information on the Study Methods

S1.1 Examples for Coding of Article/Study Types

S1.2 Assessed Background Information of Most Productive Researchers in the Survey Study

S1.3 Data Analysis

S2: Supplemental Results

S2.1 Role of the Last Author

S2.2 Main Supervisor(s) of Most Productive Researchers

S2.3 Inspiring Role Models of Most Productive Researchers

S2.4 Institutional Employment of Most Productive Researchers

**S1.1 Examples for Coding of Article/Study Types**

| **Table S1.1 Examples for Coding of Article/Study Types.** | |
| --- | --- |
| Article/study type | Example article |
| Experimental article | Cogliano, M., Bernacki, M. L., & Kardash, C. M. (2021). A metacognitive retrieval practice intervention to improve undergraduates’ monitoring and control processes and use of performance feedback for classroom learning. *Journal of Educational Psychology, 113*(7), 1421–1440. https://doi.org/10.1037/edu0000624 |
| Correlational article | Dumas, D. (2018). Relational reasoning and divergent thinking: An examination of the threshold hypothesis with quantile regression. *Contemporary Educational Psychology, 53*, 1–14. https://doi.org/10.1016/j.cedpsych.2018.01.003 |
| Case study/interview study | Fong, C. J., Flanigan, A. E., Hogan, E., Brady, A. C., Griffin, M. M., Gonzales, C., Garcia, A. J., Fathi, Z., & Robinson, D. H. (2022). Individual and institutional productivity in educational psychology journals from 2015 to 2021. *Educational Psychology Review, 34*, 2379–2403. https://doi.org/10.1007/s10648-022-09704-2 |
| Mixed method study | Rutherford, T., Liu, A. S., & Wagemaker, M. (2021). “I Chose Math Because…”: Cognitive interviews of a motivation measure. *Contemporary Educational Psychology, 66*, 101992. https://doi.org/10.1016/j.cedpsych.2021.101992 |
| Theoretical article | Chinn, C. A., Barzilai, S., & Duncan, R. G. (2020). Disagreeing about how to know: The instructional value of explorations into knowing. *Educational Psychologist, 55*(3), 167–180. https://doi.org/10.1080/00461520.2020.1786387 |
| Review article | Bardach, L., Klassen, R. M., & Perry, N. E. (2022). Teachers’ psychological characteristics: Do they matter for teacher effectiveness, teachers’ well-being, retention, and interpersonal relations? An integrative review. *Educational Psychology Review, 34*(1), 259–300. https://doi.org/10.1007/s10648-021-09614-9 |
| Meta-analysis with or without a systematic review | Cartiff, B. M., Duke, R. F., & Greene, J. A. (2021). The effect of epistemic cognition interventions on academic achievement: A meta-analysis. *Journal of Educational Psychology, 113*(3), 477–498. https://doi.org/10.1037/edu0000490 |
| Method article | Jansen, M., Lüdtke, O., & Robitzsch, A. (2020). Disentangling different sources of stability and change in students’ academic self-concepts: An integrative data analysis using the STARTS model. *Journal of Educational Psychology, 112*(8), 1614–1631. https://doi.org/10.1037/edu0000448 |
| Introduction to a special issue | Lajoie, S. P., Pekrun, R., Azevedo, R., & Leighton, J. P. (2020). Understanding and measuring emotions in technology-rich learning environments. *Learning and Instruction, 70*, 101272. https://doi.org/10.1016/j.learninstruc.2019.101272 |
| Commentary | Schunn, C. D. (2017). Building from in vivo research to the future of research on relational thinking and learning. *Educational Psychology Review, 29*(1), 97–104. https://doi.org/10.1007/s10648-016-9384-0 |
| Book review | Flanigan, A. E. (2022). Book review: SOAR to college success and beyond. *Educational Psychology Review, 34*, 1859–1864. https://doi.org/10.1007/s10648-022-09681-6 |
| Other (Category for articles that we could not classify into the above-mentioned categories.) | – |
| *Note.* A dash (–) indicates that no study of this type was identified. | |

**S1.2 Assessed Background Information of Most Productive Researchers in the Survey Study**

**Table S1.2 Demographic, Academic and Other Background Information Assessed in the Survey Study.**

**S1.3 Data Analysis**

To analyze the data from the survey study (i.e., data cleaning, calculation of descriptive statistics, mean differences with 95% confidence intervals [CI], and Chi-square tests), we used the statistical software R (R Core Team, 2023) and the R packages “janitor” (Version 2.2.0; Firke, 2023), “psych” (Version 2.3.9; Revelle, 2023), and “tidyverse” (Version 2.0.0; Wickham et al., 2019). To produce the figures, we used the R packages “ggplot2” (Version 3.4.4; Wickham, 2016), “ggsankey” (Version 0.0.99999; Sjoberg, 2023), “rnaturalearth” (Version 0.3.4; Massicotte & South, 2023), and “sf” (Version 1.0-14; Pebesma & Bivand, 2023).

**S2.4 Role of the Last Author**

Table S2.4 shows the distribution of views on the role of the last author in a scientific publication. Most researchers (35%) consider the last author position to be the second most important role, surpassed only by the first author. In contrast, 27% consider it to be the least important, and an equal number (16%) either consider it to be of varying importance or consider it to be the position of the senior author of the project. In addition, less common opinions are detailed in the table. Thus, even among highly productive scholars, there is no consensus on the role of the last author position; its importance is likely to vary with context.

| **Table S2.4 Views on the Role of the Last Author in a Publication.** | |
| --- | --- |
| Role of the last author | Percentage |
| Second most important author (after first author) | 35 |
| Least important author | 27 |
| Senior lead author | 16 |
| It varies | 16 |
| Most important author | 1 |
| Second and last author are both second most important author | 1 |
| Third most important author | 1 |
| Equal importance to all other authors if author list is alphabetically sorted | 1 |
| I prefer not to answer | 1 |

**S2.2 Inspiring Role Models of Most Productive Researchers in Top-Tier, Broad-Scope Educational Psychology Journals From 2017 to 2022**

Table S2.1 shows the frequency with which inspirational role models were named by participants in the additional survey (n = 65). By far the most frequently named inspirational role model was Jacquelynne S. Eccles with 16 votes, followed by Patricia A. Alexander (9 votes), Herbert W. Marsh (5 votes), Allan Wigfield and John Sweller (4 votes each), Oliver Lüdtke, Richard E. Mayer, and Tamara van Gog (3 votes each), among others. Three individuals reported having no inspirational role models.

| **Table S2.2 List of Inspiring Role Models Named by Most Productive Researchers.** | |
| --- | --- |
| Name | Frequency of nomination |
| Jacquelynne S. Eccles | 16 |
| Patricia A. Alexander | 9 |
| Herbert W. Marsh | 5 |
| Allan Wigfield | 4 |
| Carol Dweck | 4 |
| John Sweller | 4 |
| Oliver Lüdtke | 3 |
| Richard E. Mayer | 3 |
| Richard Ryan | 3 |
| Tamara van Gog | 3 |
| None | 3 |
| Alexander Renkl | 2 |
| Bernard Weiner | 2 |
| Florian Schmiedek | 2 |
| Jens Möller | 2 |
| Jeroen van Merriënboer | 2 |
| Judith Harackiewicz | 2 |
| Katharina Scheiter | 2 |
| Mareike Kunter | 2 |
| Andrew Elliot | 1 |
| Andrew Martin | 1 |
| Angela Ittel | 1 |
| Anne Frenzel | 1 |
| Bill McKeachie | 1 |
| Clark Chinn | 1 |
| Danielle McNamara | 1 |
| David Kenny | 1 |
| Deanna Kuhn | 1 |
| DeLeon Gray | 1 |
| Duane Shell | 1 |
| Edward Deci | 1 |
| Ellen Usher | 1 |
| Fred Paas | 1 |
| Ginger Berninger | 1 |
| Helen Watt | 1 |
| Helma Koomen | 1 |
| Jeff Greene | 1 |
| Jennifer Crocker | 1 |
| *(continues)* | |
| **Table S2.2 (continued)** | |
| Name | Frequency of nomination |
| Jessica DeCuir-Gunby | 1 |
| John Dunlosky | 1 |
| Jon Hilpert | 1 |
| Jürgen Baumert | 1 |
| Karen Harris | 1 |
| Kathryn Wentzel | 1 |
| Lisa Linnenbrink-Garcia | 1 |
| Markus Hasselhorn | 1 |
| Martin Brunner | 1 |
| Merl Wittrock | 1 |
| Mieke Brekelmans | 1 |
| Mimi Bong | 1 |
| Nikol Rummel | 1 |
| Penny Van Bergen | 1 |
| Peter Bryant | 1 |
| Philip D. Parker | 1 |
| Rainer Bromme | 1 |
| Reinhard Pekrun | 1 |
| Richard Wagner | 1 |
| Roxana Moreno | 1 |
| Shaaron Ainsworth | 1 |
| Shui-fong Lam | 1 |
| Stephan Schwan | 1 |
| Stuart Karabenick | 1 |
| Susan Goldman | 1 |
| Terezinha Nunes | 1 |
| Theo Wubbels | 1 |
| Thomas Goetz | 1 |
| Tim Mainhard | 1 |
| Toon Cillessen | 1 |
| Ulrich Trautwein | 1 |

**S2.3 Main Supervisor(s) of Most Productive Researchers**

Table S2.3 shows the frequency with which supervisors were named in the additional survey (*n* = 71). Günter Daniel Rey was the most frequently named supervisor (*n* = 3), followed by Patricia A. Alexander, Jürgen Baumert, Olaf Köller, Herbert W. Marsh, Andrew J. Martin, Jeroen van Merriënboer, Ulrich Trautwein, and Bernard Weiner (*n* = 2 each). Most respondents reported one main supervisor, 8 respondents reported two main supervisors. A total of 69 individuals were named as supervisors, which indicates a wide variance of supervisors. However, an important finding is that, not surprisingly, some of the highly productive researchers were supervised by other highly productive researchers identified in this study. Examples of these connections include: Günter Daniel Rey (advisor) – Maik Beege (advisee), Steve Nebel (advisee), and Sascha Schneider (advisee), Patricia A. Alexander – Denis Dumas (advisee) and Alexandra List (advisee), Allan Wigfield (advisor) – Emily Rosenzweig (advisee), or Jens Möller (advisor) – Fabian Wolff (advisee). In some cases, high-productivity researchers were not only advisees of a high-productivity researcher, but also supervisors of high-productivity researchers themselves: Herbert W. Marsh (advisor) – Jisei Guo (advisee) and Andrew J. Martin (advisee/advisor) – Emma Burns (advisee) and Philipp D. Parker (advisee), Olaf Köller (advisor) – Marlit Annalena Lindner (advisee) and Ulrich Trautwein (advisee/advisor) – Uta Klusmann (advisee) and Hanna Gaspard (advisee), Fred Paas (advisor) – Tamara van Gog (advisee/advisor) – Vincent Hoogerheide (advisee).

| **Table S2.3 Main Supervisors of the Most Productive Researchers in Top-Tier, Broad-Scope Educational Psychology Journals From 2017 to 2022.** | |
| --- | --- |
| Name | Frequency |
| Günter Daniel Rey | 3 |
| Andrew J. Martin | 2 |
| Bernard Weiner | 2 |
| Herbert W. Marsh | 2 |
| Jeroen van Merriënboer | 2 |
| Jürgen Baumert | 2 |
| Olaf Köller | 2 |
| Patricia A. Alexander | 2 |
| Ulrich Trautwein | 2 |
| Allan Wigfield | 1 |
| Allen Parducci | 1 |
| Anat Zohar | 1 |
| Angela Ittel | 1 |
| Benjamin Nagengast | 1 |
| Catherine Snow | 1 |
| David Klahr | 1 |
| Detlev Leutner | 1 |
| Floyd Hudson | 1 |
| Frank Manis | 1 |
| Franz Weinert | 1 |
| Fred Paas | 1 |
| George Farkas | 1 |
| Heinz Holling | 1 |
| James Greeno | 1 |
| James Michael Royer | 1 |
| James P. Byrnes | 1 |
| Jari-Erik Nurmi | 1 |
| Jennifer Shapka | 1 |
| Jens Möller | 1 |
| Karl F. Wender | 1 |
| Katharina Scheiter | 1 |
| Keith Widaman | 1 |
| Kennedy T. Hill | 1 |
| Kenneth A. Kiewra | 1 |
| Kirsten Berthold | 1 |
| Klaus Schneewind | 1 |
| Lisa Linnenbrink-Garcia | 1 |
| Mareike Kunter | 1 |
| *(continues)* | |
| **Table S2.3 (continued)** | |
| Name | Frequency |
| Martin Ford | 1 |
| Matthias Nückles | 1 |
| Michael E. Martinez | 1 |
| Michel Janosz | 1 |
| Mieke Brekelmans | 1 |
| Ola Bø | 1 |
| Oliver Lüdtke | 1 |
| Paul Pintrich | 1 |
| Peter Gerjets | 1 |
| Philip H. Winne | 1 |
| Reinhard Pekrun | 1 |
| Richard E. Mayer | 1 |
| Robert J. Vallerand | 1 |
| Roger Azevedo | 1 |
| Rolf Steyer | 1 |
| Ruth Garner | 1 |
| Serge Larivée | 1 |
| Shin-ichi Ichikawa | 1 |
| Shui-fong Lam | 1 |
| Sofie Loyens | 1 |
| Stanley L. Deno | 1 |
| Steven R. Asher | 1 |
| Stuart Karabenick | 1 |
| Tamara van Gog | 1 |
| Terezinha Nunes | 1 |
| Theo Wubbels | 1 |
| Toni Falbo | 1 |
| Tony Winefield | 1 |
| Ulrich Schroeders | 1 |
| Uwe Grau | 1 |
| Vincent Aleven | 1 |

**S2.4 Institutional Employment**

| **Table S2.4 Institutions Where the Most Productive Scholars Identified in This Study Are (or Were) Employed.** | |
| --- | --- |
| Institution | Frequency |
| University of Tübingen | 7 |
| Australian Catholic University | 5 |
| Leibniz Institute for Science and Mathematics Education (IPN) | 4 |
| University of New South Wales | 4 |
| Kiel University | 3 |
| TU Dortmund University | 3 |
| University of Georgia | 3 |
| University of Maryland, College Park | 3 |
| University of Potsdam | 3 |
| Leibniz-Institut für Wissensmedien (IWM) | 2 |
| McGill University | 2 |
| University of California, Irvine | 2 |
| University of North Carolina at Chapel Hill | 2 |
| Utrecht University | 2 |
| American Institutes for Research | 1 |
| Arizona State University | 1 |
| Chemnitz University of Technology | 1 |
| Cluster of Excellence Science of Intelligence (SCIoI) | 1 |
| Concordia University | 1 |
| DIPF \| Leibniz Institute for Research and Information in Education | 1 |
| Erasmus University Rotterdam | 1 |
| Freiburg University of Education | 1 |
| Georgia Southern University | 1 |
| Institute for Educational Quality Improvement (IQB) | 1 |
| Kent State University | 1 |
| Leiden University | 1 |
| Macquarie University | 1 |
| Michigan State University | 1 |
| Purdue University | 1 |
| Ruhr University Bochum | 1 |
| The Pennsylvania State University | 1 |
| The University of Hong Kong | 1 |
| The University of Texas at Austin | 1 |
| University of Bonn | 1 |
| University of California, Los Angeles | 1 |
| University of California, Santa Barbara | 1 |
| *(table continues)* | |
| **Table S2.4 (continued)** | |
| Institution | Frequency |
| University of Delaware | 1 |
| University of Essex | 1 |
| University of Freiburg | 1 |
| University of Haifa | 1 |
| University of Heidelberg | 1 |
| University of Helsinki | 1 |
| University of Koblenz | 1 |
| University of Macau | 1 |
| University of Missouri | 1 |
| University of Oslo | 1 |
| University of Pittsburgh | 1 |
| University of Reading | 1 |
| University of Southern California | 1 |
| University of Trier | 1 |
| University of Vienna | 1 |
| University of Wisconsin-Madison | 1 |
| University of Wollongong | 1 |
| University of Zurich | 1 |
| Universtité Laval | 1 |
| Vanderbilt University | 1 |

**References**

Firke, S. (2023). *janitor: Simple tools for examining and cleaning dirty data* [R package version 2.2.0]. Retrieved from <https://CRAN.R-project.org/package=janitor>

Massicotte, P., & South, A. (2023*). rnaturalearth: World map data from natural earth* [R package version 0.3.4]. Retrieved from <https://CRAN.R-project.org/package=rnaturalearth>

Pebesma, E., & Bivand, R. (2023). *Spatial data science: With applications in R.* Chapman and Hall/CRC. <https://doi.org/10.1201/9780429459016>

R Core Team (2023). *R: A language and environment for statistical computing.* R Foundation for Statistical Computing. Vienna, Austria. <https://www.R-project.org/>

Revelle, W. (2023). *psych: Procedures for psychological, psychometric, and personality research* [R package version 2.3.9]. Northwestern University, Evanston, Illinois. Retrieved from <https://CRAN.R-project.org/package=psych>

Sjoberg, D. (2023). *ggsankey: Sankey, alluvial and sankey bump plots* [R package version 0.0.99999].

Wickham, H. (2016). *ggplot2: Elegant graphics for data analysis.* Springer.

Wickham, H., Averick, M., Bryan, J., Chang, W., McGowan, L. D., François, R., Grolemund, G., Hayes, A., Henry, L., Hester, J., Kuhn, M., Pedersen, T. L., Miller, E., Bache, S. M., Müller, K., Ooms, J., Robinson, D., Seidel, D. P., Spinu, V., ... Yutani, H. (2019). “Welcome to the tidyverse.” *Journal of Open Source Software* 4 (43): 1686. <https://doi.org/10.21105/joss.01686>
